# Input, process, and output factors contributing to quality of antenatal care services: a scoping review of evidence

**DOI:** 10.1186/s12884-022-05331-5

**Published:** 2022-12-28

**Authors:** Resham B Khatri, Tesfaye S Mengistu, Yibeltal Assefa

**Affiliations:** 1 School of Public Health, the University of Queensland, Brisbane, Australia; 2 Health Social Science and Development Research Institute, Kathmandu, Nepal; 3 College of Medicine and Health Sciences, School of Public Health, Bahir Dar University, Bahir Dar, Ethiopia

**Keywords:** Antenatal care, Quality, Health systems, Inputs, Processes, Outputs

## Abstract

**Background:**

High-quality antenatal care (ANC) provides a lifesaving opportunity for women and their newborns through providing health promotion, disease prevention, and early diagnosis and treatment of pregnancy-related health issues. However, systematically synthesised evidence on factors influencing the quality of ANC services is lacking. This scoping review aims to systematically synthesize the factors influencing in provision and utilisation of quality ANC services.

**Methods:**

We conducted a scoping review of published evidence on the quality of ANC services. We searched records on four databases (PubMed, Scopus, Embase, and Google scholar) and grey literature from 1 to 2011 to 30 August 2021. We analysed data using Braun and Clarke’s thematic analysis approach. We followed Preferred Reporting Items for Systematic Reviews and Meta-Analyses extension for Scoping Reviews (PRISMA-ScR) guideline for the review. We explained themes using the Donabedian healthcare quality assessment model (input-process-output).

**Results:**

Several inputs- and process-related factors contributed to suboptimal quality of ANC in many low and lower- or middle-income countries. Input factors included facility readiness (e.g., lack of infrastructure, provision of commodities and supplies, health workforce, structural and intermediary characteristics of pregnant women, and service delivery approaches). Processes-related factors included technical quality of care (e.g., lack of skilled adequate and timely care, and poor adherence to the guidelines) and social quality (lack of effective communication and poor client satisfaction). These input and process factors have also contributed to equity gaps in utilisation of quality ANC services.

**Conclusion:**

Several input and process factors influenced the provision and utilization of optimum quality ANC services. Better health system inputs (e.g., availability of trained workforces, commodities, guidelines, context-specific programs) are essential to creating enabling facility environment for quality ANC services. Care processes can be improved by ensuring capacity-building activities for workforces (training, technical support visits), and mentoring staff working at peripheral facilities. Identifying coverage of quality ANC services among disadvantaged groups could be the initial step in designing and implementing targeted program approaches.

**Supplementary Information:**

The online version contains supplementary material available at 10.1186/s12884-022-05331-5.

## Introduction

Pregnancy and childbirth represent a period of a heightened risk of morbidity and mortality for both women and their unborn babies [1]. Global estimates show that more than 800 women die every day due to pregnancy and childbirth complications, where 99% of maternal deaths are from low and lower- or middle-income countries (LMICs), especially in sub-Saharan Africa (SSA) and South Asia (SA) [2, 3]. Moreover, while the Maternal Mortality Ratio (MMR) has decreased by up to 40% in the last three decades in SSA and SA; still,  MMR has remained unacceptably high in those regions [4].

Antenatal care (ANC) visits comprise high-impact interventions with the potential to prevent and reduce maternal and perinatal morbidity and other severe adverse outcomes, including perinatal deaths. Yet, ensuring access to quality ANC and increasing its utilisation have long been challenges in many LMICs with stubbornly high MMR and NMR, where health systems are weak and fragile [5–7]. Globally, there has been increasing access to maternal and newborn health (MNH) visits (such as 4 ANC visits or childbirth assisted by skilled birth attendants (SBAs)). But regional inequity exists in utilisation of those MNH visits. For instance, the global average uptake of 4ANC visits was 75%, with almost universal coverage in North America and Europe; in contrast, only 52% of women in the SSA received MMR at the highest [8, 9].

Furthermore, in many LMICs maternal, and rate of reduction of perinatal deaths is slow compared to the increase in routine MNH visits [10, 11]. Inadequate access to quality ANC services is one of the reasons behind slow reduction of preventable maternal and perinatal deaths. Conversely, the provision of high-quality ANC ensures the delivery of lifesaving interventions such as birth preparedness and complication readiness for early diagnosis and treatment of pregnancy-related complications [12]. To ensure optimum care during pregnancy, the World Health Organisation (WHO) recommends every pregnant woman have a minimum of four ANC visits, with the first antenatal visit in the first trimester of pregnancy [13]. Furthermore, informed by a study by Downe and colleagues published in 2016 [14], the WHO recommended eight or more ANC visits for women with positive pregnancy experiences [15]. Furthermore, the WHO also emphasises and includes the respect and dignity of service users as an essential component of quality of care at pregnancy and maternity period [16].

Routine ANC visits encourage women to take recommended interventions for healthy pregnancies and newborns and reduce adverse pregnancy outcomes. For example, evidence suggests that women who complete recommended ANC visits are more likely to give birth at a health facility and complete postnatal care (PNC) and give childbirth assisted by SBAs compared to a pregnant woman without ANC visits [17, 18]. In addition, uptake of recommended ANC interventions requires better health facility readiness (e.g., provision of essential medicines, trained health workforces), delivery of interventions, and uptake by disadvantaged population groups [19, 20].

Measurement of quality of care requires a wide range of information on health facility readiness (inputs), processes of care and users’ experiences (processes), and effects of care (outputs), making quality of care a complex concept. The Donabedian model of quality health care conceptualizes quality from inputs, processes, and outcome perspectives [21]. Input refers to structural *quality* (infrastructure, trained workforces), *care process* covers the technical quality (uptake of clinical interventions) and *social quality* (satisfaction, communications), and *outcomes* (e.g., improved health status and effectiveness). For example, the processes of care of quality ANC assessment refers to adequate care (e.g., completion of at least four ANC visits), timely care (first visit within the first month of pregnancy), skilled care (provided by SBAs), and sufficient care (receiving recommended interventions) [11]. A large body of literature focuses on adequate care (completion of 4ANC visits) as the indicator of quality ANC; however, only completion of recommended visits does not guarantee timely and sufficient uptake of recommended interventions [22, 23]. Furthermore, studies lack what factors influence the provision and utilisation of quality ANC services. This scoping review synthesised evidence regarding the contributing factors of high-quality ANC services.

## Methods

We conducted a scoping review of published evidence reporting the quality of ANC. The review was conducted following the Preferred Reporting Items for Systematic Reviews and Meta-Analyses extension for Scoping Reviews (PRISMA-ScR) guideline (Appendix, Table S1) [24].

### Data sources and search strategy

We searched four electronic databases (PubMed, Scopus, Embase, and Google scholar) and grey literature for studies describing quality of ANC quality. This was followed by complementary searches (e.g., citation searches of selected studies) and Google searches to locate further eligible articles that were not identified in the database searches. The keywords used in the search strategy were built on two key concepts (ANC and quality) and tailored to each database (Appendix, Table S2). Boolean operators and truncations were varied depending on the database.

### Inclusion and exclusion criteria

The search included articles published in English from January 1 2011 to 30 August 2021. In addition, we included review studies published before 30 December 2010. No country-related limitations were applied. We included all relevant studies that dealt with ANC and its quality measures regardless of the study design (quantitative, qualitative, mixed methods, reviews, reports, and secondary data analysis). Data were managed using EndNote X9.0 software. We selected studies following the PRISMA-ScR guideline [24] and referenced previous scoping review studies [25, 26] where quality grading of studies was not assessed and was not a criterion for inclusion. In addition, we included studies considering the population (ANC services users, concept (quality) and content (input-process-output factors) of the study that can answer our review question rather than the quality of studies included in our review [27, 28].

### Data extraction, analysis, and synthesis

Two reviewers conducted screening and full-text review. The first author (RBK) screened studies based on title, abstract and full text. The second author (TSM) checked the eligibility of selected studies. Data were extracted by the first author (RBK) and double-checked by the second author (TSM). The last author (YA) verified the search strategy, screening process and selected studies in the review. Any disagreements were resolved by discussion. We used the inductive thematic analysis of data using the Braun and Clarke thematic analysis approach [29]. An inductive thematic analysis approach involves the generation of themes from data rather than preconceived themes that researchers expect to get from the data or based on theory or existing knowledge [30]. Braun and Clarke’s thematic analysis  approach includes the following six steps for qualitative data analysis: (1) familiarising with data; (2) generating initial codes; (3) searching for themes; (4) reviewing themes; (5) defining and naming themes; and (6) producing the report. First, a data extraction sheet was developed covering each article’s author, year, country, types of study, the main concept of ANC quality, and main findings (Appendix, Table S3). Then, the important ideas (codes) were extracted and read again. Similar data extract was grouped to identify subthemes. Subthemes with similar ideas were grouped again into themes, analysed, synthesised, and explained in paragraph form. Finally, generated themes were mapped and explained using the Donabedian model of quality of health care (input-process-output) [21]. Themes under inputs included organisational factors, users’ characteristics, and service delivery approaches. Themes under processes of care included technical quality and perceived quality of ANC services. Themes under outputs included equity gaps, population-level coverage of quality ANC, and effectiveness of ANC interventions.

## Results

**Fig. 1:**
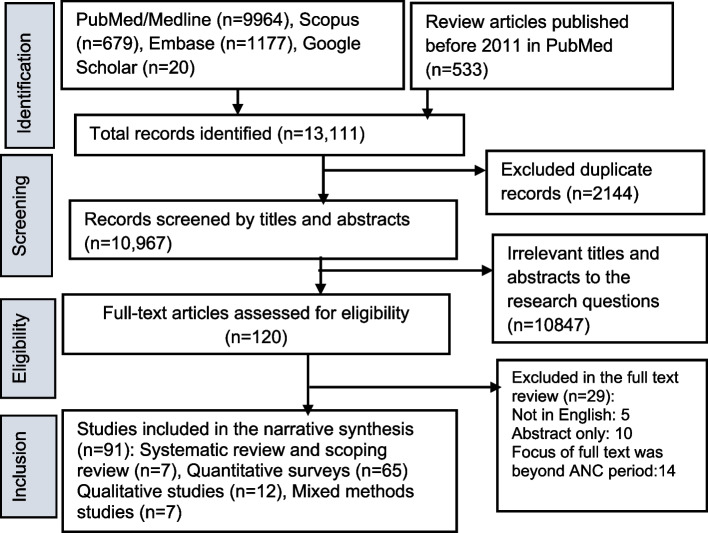
PRISMA-ScR flowchart showing the selection of studies in the review

Figure 1 presents the study screening process, selection, and reasons for exclusion. We extracted studies from PubMed, Scopus, Embase, and Google Scholar since 2011, while we included review studies only before 1 January 2011. We included eighty-nine articles in the final review.

### Domains, themes, and factors (facilitators and barriers) of ANC quality

Of 91 studies, 60 described inputs and processes-related factors and 20 factors related to outputs. Some studies also described factors in multiple domains (Table 1).

**Table 1:** Themes and factors contributing to quality of ANC services

Domains	Themes	Facilitators	Barriers	Countries
Inputs	Organisational factors	Better structural quality (inputs) [31], better infrastructure and health systems [32–34]	Poor readiness for ANC services, lack of certain services and provisions [35–37]	Nepal, Sub-Saharan Africa (SSA), Benin, Ghana, Ethiopia, Kenya, SSA, Lao People Democratic Republic, Pakistan
	Supplies of commodities	Better supply of essential commodities [38, 39], laboratories facilities [40, 41]	Stock-out of medical supplies [42–45], lack of facilities [35, 46], late reimbursement of funds, lack of policy definition, out of pocket payment [33, 37, 40, 44, 45, 47–50]	Ghana, Ethiopia, Jordan, Tanzania, LMICs, SSA,
	Trained health workforces	Trained and skilled providers [31, 51, 52], better qualification and training of staff [32, 40], staff supervision [31], and flexible time for appointment [41]	Lack of feedback, motivation and competence, shortage of staff, lack of human resources, limited skills, and non-availability [42, 47, 49], heavier workload, inequitable distribution, unavailability of skilled providers, lack of privacy and confidentiality [45, 47, 49, 53, 54]	Nepal, Rwanda, SSA, Ethiopia, Jordan, Lao PDR, Tanzania, Pakistan, Namibia,
	Structural determinants	Education, SES, job and income [11, 38, 52, 55–68] [69]. [40], middle age and lower parity [38, 56, 57, 59, 60, 62, 66], settled residence [57]	Poor socioeconomic status, low education, low family income, Indigenous groups, unemployed, [52, 68, 70–75]	Malawi, Mexico, Pakistan, Iran, LMICs, Camron, Ethiopia, Tanzania, Nigeria, Nepal, Zambia, East African countries, Egypt, Myanmar, India
	Intermediary determinants	insured women [11, 76], women with higher empowerment and decision-making power [52, 58, 65], urban areas [38, 52, 56, 58–60, 62, 63, 66, 68], attended big hospitals and private HFs [52, 55, 56, 59, 62–64, 71, 77], non-smokers women, intended pregnancy or first baby, networking women, received the maternal and child health handbook, previous history of complication [52, 60, 61, 65, 68, 73, 78, 79], media exposure and women empowerment [52, 61, 80]	Rural and remote locations, slum areas [64, 65, 70, 75, 80], high birth order, low age at marriage and childbirth (e.g., adolescents), short time intervals, old age, unmarried [52, 65, 68, 70, 72–75, 80], inadequate ANC, poor priority and awareness, late recognition of pregnancy, single parent, smoker, unplanned place of delivery, without insurance [74, 81, 82]	Kenya, Mexico, LMICs, Nepal, Ghana, Pakistan, Ethiopia, Tanzania, Nigeria, Cameroon, East African countries, Brazil, Malawi, Egypt, Kenya, Myanmar, South Asia, Madagascar, Oman, India, High income countries, Bangladesh, Brazil
	Services delivery approaches	Cultivating quality care at public facilities, supportive supervision of providers [40, 83], group ANC approach, collaboration with local government clinics [84], task shifting and training of care providers [85], home visitation, community mobilization, training of CHWs, logistical support, monitoring and documentation [84, 85], digital technology [86]	Poor knowledge and attitude, lack of decision-making autonomy, poor empowerment, poor access to media exposure [57, 68, 80, 81, 87], superstitions around pregnancy [81], client awareness [49]	LMICs, Ethiopia, Nepal, Bangladesh, south Asia, East African, Afghan, Uganda Pakistan,
Process of care	Skilled care	Interaction with providers, privacy, attentiveness of providers, trained personnel, extended time, hospital care [38, 63, 75, 78, 88], birth preparedness, and counselling [88]	Improper registration, history taking and assessment, lack of counselling on prevention and treatment, and poor client-provider interactions [49, 66, 88]	India, Tanzania, Ghana, Oman, Nigeria, Ethiopia, Pakistan,
	Timely care	Early initiation of 1^st^ ANC visit [63], longer appointment [89], ANC in the first trimester [59, 64, 70, 78, 90, 91]	No early visit, high birth order [37, 59, 92], short time care [47], high dropout in subsequent visit [49], late first ANC visit [79]	Nigeria, Ghana, LMICs, Ethiopia, Cameroon, Peru, Zambia, Lao PDR, Pakistan, Madagascar
	Adequate care	High ANC visits [37, 89, 93, 94] better health promotion in counselling (e.g., healthy eating, danger signs) [39, 46, 59, 79]	Low uptake of ANC interventions [60, 65, 75, 78, 80, 95], low quality and coverage gaps [11, 46, 57, 59, 68, 70, 77, 89, 92–94, 96], ANC visit but no uptake of interventions [63, 90, 91, 94, 97], low clinical quality [36, 66], low uptake of technical interventions [36]	Zambia, Mexico, Ghana, Cameroon, Madagascar, Ethiopia, Zambia, Oman, India, Nepal, South Asian countries, Afghan, Brazil, Mexico, Sierra Leone, Nigeria, East African Countries, Peru, Kenya
	Adherence to guideline	Adherence to some guidelines (tetanus toxoid, lifestyle, blood test) [62, 91, 94, 98, 99], counselling and iron folate uptake [100]	Poor adherence to local guidelines and standards [44, 67, 82, 90, 98], evidence-based guidelines [43, 44, 50, 63, 82, 97, 99–104]	Peru, Australia, Tanzania, Ethiopia, Bangladesh, LMICs, Brazil, Zambia, eight countries of SSA and SA, Indonesia, west and central Africa, Ghana, Nigeria
	Effective communication	Communication between providers and users [103], interpersonal communication, and training on communication skills [41, 55, 105]	Ineffective communication behaviour and attitude, lack of privacy, unequal treatment to clients [53, 54, 69, 87], poor awareness of complications, lack of understanding of tests and medicine, cost and quality [41, 69, 87], inadequate communication skills of providers [53, 83]	Ghana, Malawi, Jordan, Kenya, Namibia, Uganda, LMICs
	Client satisfaction	Providers, explanation of procedures, consent seeking, encouragement to ask questions, confidentiality, and good interpersonal relations [41, 55, 106–108]	Poor satisfaction with ANC services [106, 109, 110], lack of privacy, discrimination, being left unattended, providers’ attitude, delayed and inadequate care, physical abuse, inappropriate position in the examination, lack of privacy, negative assumptions and disregard for mothers’ options in care, long waiting time [54, 95, 105, 107–110]	Malawi, Jordan, Ethiopia, Rwanda, Myanmar, Kenya, Zambia,
Outputs	Quality- adjusted coverage	High contact coverage of 4ANC visits [67, 111, 112]	Lower average coverage for care content and quality-adjusted coverage [67, 71, 73, 94, 101, 111–113]	Ethiopia, Zambia, Rwanda, Myanmar, LMICs, Egypt
	Equity gaps	High coverage among some privileged groups [35, 36]	Equity gaps within and between countries [101, 113, 114], regional inequity [77], regional and ethnicity-based inequity [11, 115], SES and regional inequity [56, 58, 68, 75, 112], [75]	Ethiopia, Kenya, LMICs, SSA, eight countries, Brazil, Mexico, East African countries, Rwanda, India, Pakistan,
	Effectiveness	ANC quality was inversely associated with preterm birth and neonatal mortality [116, 117]	Low mortality reduction for people with minorities [115]	LMICs, Mexico

### Inputs

Inputs-related factors (themes) contributing to quality of ANC included health system factors (e.g., health workforce, commodities, health facility readiness), structural and intermediary characteristics of users, and approaches to ANC service delivery.

### Organizational factors of quality ANC

Better health facility readiness improves the provision of quality ANC. Women utilized better quality ANC services if health facilities and better structural quality (inputs) in Nepal [31]. Characteristics of better facility readiness include the availability of ANC guidelines and procedures, supply of central electricity, information, organization of services, community health planning, and patient reception and interpersonal communication [32–34].

In many LMICs, health facilities had poor readiness for ANC services [35–37]. Factors of poor readiness were rural and remote locations of facilities, insufficient resources (equipment, supplies), poor organization of facilities, lack of certain services and provisions (health education sessions), lack of policy on human resources, late reimbursement of funds, and out of pocket payment [33, 37, 40, 44, 45, 47–49]. In addition, poor correlation exists between infrastructure of health facilities and evidence-based care that has provided a varying level of service quality [50].

### Supplies of commodities

Health commodities were necessary for the quality of ANC services. Availability of commodities for better quality included iron-folate, tests for syphilis and HIV, diabetes screening, ultrasound, fetal heart sound, maternal weight, blood pressure, tetanus toxoid vaccine, and essential drugs [38, 39]. In addition, laboratory facilities (blood tests, equipment, and devices) are supported to improve quality of ANC services [40, 41]. However, a shortage of equipment, commodities, and medicines (reagents and consumables, essential drugs, and medical supplies) hindered the provision of recommended care [42–45]. For example, in Ethiopia, there was a lack of the availability of tracer items (e.g., tetanus vaccination, folic acid, iron, and test items for syphilis) for ANC services [35, 46].

### Trained health workforces

The availability of trained health workforces (types, skills, and qualifications) could improve the quality of ANC services. Factors of better-quality ANC services included service provided by nurses/midwives, availability of pregnancy-related information, and skilled healthcare providers [31, 51, 52]. Other factors were cultivating quality care at public facilities, training on improving communication skills, and supportive supervision of providers [40, 83]. The Quality of ANC was optimal if providers had high qualifications and experience and priority of ANC, continued attention of providers and information on clinical care [32, 40]. In addition, women utilized better quality ANC if the service was provided by supervised staff in Nepal [31]. The availability of female providers and flexible appointment times in Jordan [41]. Nonetheless, shortage of staff, limited skills, and poor provision of motivation, competence, and feedback were factors of poor quality of ANC services [42, 47, 49]. In addition, heavier workload, inequitable distribution of staff, lack of privacy and confidentiality, and ineffective communication skills (e.g., providers’ behaviour, providers’ poor technical preparedness and different attitudes) were factors of good-quality ANC services [45, 47, 49, 53, 54].

### Structural characteristics

Social determinants of service users also influence quality of ANC services. For instance, women received better quality ANC if they had education (husband and wife), higher socioeconomic status (SES), were from advantaged ethnicity, had husbands with paid jobs, and had high family income [11, 38, 52, 55–68]. Additionally, literate, employed, and wealthiest women received an ultrasound service in Kenya [69]. High maternal education was positively associated with identifying pregnancy-related danger signs [40].

Women with advantaged groups (high SES, high level of education) received ANC earlier in the pregnancy [64, 92]. Pregnant women received better quality ANC care if they were 30–40 years old and had low parity [38, 56, 57, 59, 60, 62, 66]. In Iran, women of Afghan Refugees backgrounds received optimal quality ANC if they had a more extended stay and with legal status (verified status) [57]. Additionally, insured women received adequate ANC or could pay for private health care for better quality ANC services [11, 76]. Women’s empowerment and decision-making power contributed to better quality services [52, 58, 65]. Women living in urban areas or short distances to HFs were associated with the uptake of better-quality services [38, 52, 56, 58–60, 62, 63, 66, 68]. Furthermore, if women attended big hospitals and private facilities receive optimum quality services during their pregnancy [52, 55, 56, 59, 62–64, 77]. In Egypt, two-thirds of women visited four more ANC visits in private hospitals, while less than 50% of women completed in public facilities [71].

However, poor completion of ANC and interventions was low if women belonged were from disadvantaged groups (low SES, low education, unemployed status, low family income, and Indigenous women) [52, 68, 70–75]. In addition, women received poor quality ANC if women were from rural residences and slum areas [64, 65, 70, 75, 80], had higher birth order, low age at marriage and childbirth (e.g., adolescents), unmarried, short time intervals of pregnancies, and old age [52, 65, 68, 70, 72–75, 80].

### Intermediary characteristics

Women received better quality ANC if women were non-smokers, intended pregnancy or first baby, networking women, insured women, received the MCH handbook, and had a previous history of infection or anaemic conditions [52, 60, 61, 65, 68, 73, 78, 79]. Media exposure and women empowerment was positively associated with adequate ANC services [52, 61, 80]. Health education sessions for women were positively influenced to identify danger signs in their pregnancy [40, 83].

Nevertheless, women received poor quality ANC if they had poor priority and low awareness, adolescent group, high parity, late recognition of conception, single parent, smoker women, unplanned place of delivery, and uninsured [74, 81, 82]. There was poor access to quality ANC if women had poor knowledge and attitude, a lack of decision-making autonomy, poor empowerment, and media exposure [57, 68, 80, 81, 87]. Many superstitions around pregnancy in rural Bangladesh received poor quality services, and women sought late ANC visits, and they sought care from traditional care [81].

### Service delivery approaches

Implementation of innovative approaches also increases the quality of ANC services. For example, gestational matched and stable group ANC approach, collaboration with local government clinics, home visitation, and community mobilization improved the quality of ANC services [84, 85]. Task shifting and training of midwives and Community Health Workers (CHWs) improved ANC visits and uptake of health interventions (e.g., uptake of tetanus toxoid, early uptake of breastfeeding, reduction of referral and hospital admission) [85]. Training of CHWs, training and logistical support, and monitoring and documentation were some strategies for ANC quality improvement toward better MNH outcomes [84, 85]. Use of digital tools can address the demand-side factors through increasing timely access and content of ANC, health promotion, and consumer awareness [86].

Additionally, digital systems can strengthen supply-side factors by organisation and delivery of services, spatial mapping of access gaps, continuity of patient records and data-supported decision-making, performance measurement, reduction of clinical errors, care coordination, and integration and engineering collaborative innovation [86]. For example, women’s empowerment (decision-making on health seeking, financing and information) has increased the uptake of quality maternity care in Guinea [118]. In contrast, a lack of client awareness, self-empowerment and healthcare decision-making was associated with low coverage in Punjab [49].

### Processes of care

In the process domain, themes explained the technical and perceived quality of care (skilled care, timely care, adequate care and adherence to guidelines, effective communications, and client satisfaction).

### Skilled care

Skilled care providers are important for the delivery of good quality clinical care. Factors contributing to better-skilled care were interaction with providers, privacy in consultation, attentiveness of providers, trained personnel, extended time in health care visits, and services received in hospitals [38, 63, 75, 78, 88]. In addition, providers followed the birth preparedness and complication readiness plans (e.g., counselling on HIV) in Tanzania [88]. However, factors of inadequate skilled care, improper registration, history taking and assessment, lack of counselling on prevention and treatment, including danger signs, and poor client-provider interactions [49, 66, 88].

### Timely care

Timely care of ANC is vital in pregnancy and childbirth. For instance, early initiation of ANC visits and completion of 4ANC visits as per protocol [63]. In Ghana, women had higher odds of eight ANC contacts if providers spent longer the appointment (more than 20 min) time in the first ANC visit, and women received home visits from skilled providers [89]. Nonetheless, despite universal 4ANC visits and receiving care from trained personnel in Oman, only 75% attended ANC visits in their 1st trimester [78]. There was late initiation of ANC visits (ANC in the first trimester) in many LMICs [59, 64, 70, 90, 91]. Women had late initiation of ANC visits if they had high birth orders in Camron [59]. One in three women received ANC within the first trimester of pregnancy in countries of SSA [37, 92]. Each woman’s average consultation time was five minutes in Lao PDR [47]. High dropout was observed in the MNH continuum with first ANC (55.9%) to subsequent visits (32.9%) in Punjab [49]. More women attended their first ANC visit in Madagascar’s second trimester of pregnancy [79].

### Adequate care

Early initiation and adequate ANC visits contributed to counselling on healthy eating, promoting maternal and fetal health, intended pregnancy, or awareness of previous conditions (e.g., anaemia) [39, 59, 79]. Uptake of adequate ANC improved utilization of institutional delivery [46]. Nonetheless, the uptake of recommended 4ANC visits was low in many LMICs, including low uptake of recommended interventions [60, 65, 75, 78, 80, 95]. In Nepal, a study revealed that two-thirds of women who completed 4ANC visits; only 21% received recommended interventions [65]. In Zambia, despite high first ANC visit, only three in five attended 4ANC visits, and one-third received optimal quality service [37]. Despite high first ANC visit, high quality and coverage gaps occurred, and women low proportion of women completed 4ANC visits [89, 93, 94] [57, 94]. In Latin America (e.g., Brazil and Mexico), there was low uptake of 4ANC visits and recommended interventions [11, 77]. A similar pattern of poor uptake of ANC interventions was observed in Africa [46, 59, 68, 70, 92, 96], including in Ethiopia [66] and Kenya [36], and Asia (Malaysia, Pakistan, Iran) [72, 83, 117]. Only completion of recommended visits did not guarantee utilisation of recommended ANC interventions [63, 90, 91, 94, 97]. In Kenya in 2015, less than one in five (17%) women received the minimum standard (0.75 of 1.00) of quality ANC care [36].

### Adherence to guidelines

Studies reported some adherence to guidelines and standards. For instance, healthcare providers followed guidelines on health education, iron supplementation, measurement (blood pressure and body weight), tetanus toxoid immunisation, screening guidelines (blood glucose), identification of complications, and lifestyle modification interventions [62, 91, 94, 98, 99]. In addition, there was increased compliance with the supplementation of iron-folate during pregnancy and counselling and awareness of anaemia [100].

Some studies reported poor adherence to local guidelines for follow-up of highly prevalent problems (e.g., anaemia, smoking, unitary tract infections, and sexually transmitted infections), conjunctiva check anaemia, tests (blood glucose and urine protein, venereal diseases), and information on pregnancy complications [44, 67, 82, 90, 98]. Few studies reported low adherence to evidence-based guidelines of basic clinical care in pregnancy that cover physical examination (e.g., oedema, body weight, fundal height), tests (blood and urine), lack of appropriate history taking, and administration of iron tablets [50, 99, 101, 102]. In some settings, there was inadequate counselling and education sessions on malaria prophylaxis, iron supplementation, attention to client’s wellbeing, venereal diseases test, HIV testing, and blood grouping [43, 44, 63, 82, 97, 100, 103, 104].

### Effective communications

Communication between providers and users is essential for the delivery of ANC services. Providers’ better interpersonal and communication skills contributed to confidentiality, privacy, interpersonal relations, and service delivery procedures, and perceived better-quality services [41, 55, 103, 105]. Training and continuing education use effective materials, rewards, and feedback systems to improve providers’ communication skills [53, 83]. Nevertheless, health workforce factors of ineffective communication that influenced quality of care in pregnancy were lack of interaction with providers and providers, lack of explanation of procedures, poor linking the procedures with preventive information, lack of respect with clients, behaviour and attitude of providers, lack of privacy, unequal treatment to clients [53, 54, 69, 87]. Demand side factors of ineffective communication were poor awareness of pregnancy complications, lack of understanding of tests and medicine, perception of poor quality in public hospital services for complicated pregnancies, and high cost and quality at private hospitals [41, 69, 87].

### Clients’ satisfaction

Client satisfaction is an important component of the social quality of ANC services. Studies reported several factors, primarily supply side of health systems. These factors were counselling on laboratory (test) services, supplementation of commodities (e.g., iron), information on foetal movement and dangerous signs, respectful maternity care, planning pregnancy, tailored care for mothers and foetus based on individual needs, functional patient-provider relationships, acknowledgement of the need social context of clients and culturally sensitive care, self-introduction by providers, explanation of procedures, consent seeking, encouragement to ask questions, confidentiality and good interpersonal relations [41, 55, 106–108]. Demand side factors of improving client satisfaction were long consultation time and previous ANC visits [41, 55, 106–108].

Studies reported poor satisfaction with ANC services in many IMICs [106, 109, 110]. Factors of poor client satisfaction were lack of privacy, discrimination based on specific attributes and being left unattended, providers’ attitude, delay in providing care, inadequate care, unavailability of skilled providers, physical abuse, not being allowed to choose a position in the examination, lack of privacy mothers’ multiple complications, providers’ negative assumptions and disregard for mothers’ options in care, and long waiting time [54, 95, 105, 107–110].

### Outputs

Themes under output of quality ANC included quality-adjusted coverage, equity, and effectiveness.

### Quality-adjusted coverage

Studies showed poor effective coverage of health services. Despite high 4ANC visits, the quality-adjusted coverage or average coverage of the content of care was low [67, 71, 73, 94, 101, 111–113]. Countries had lower average coverage for care content than 4ANC visits, but average coverage of uptake of recommended interventions in the routine visit was low in many LMICs [94]. For example, in 2017, the completion of 4ANC visits in Haiti was 65%, while effective coverage or quality-adjusted coverage ( average uptake of recommended interventions in 4ANC visits) was 29% [101]. Effective coverage of ANC for pregnant women was substantially lower than crude service coverage due to major deficiencies in care quality [101]. In Ethiopia in 2016, the average coverage of 4ANC visits was 62% when adjusting the crude coverage by service quality; the mean quality-adjusted coverage of ANC services was 22% [111].

### Equity gaps

There were equity gaps in utilising ANC services within and between countries [101, 113]. For instance, regional and socioeconomic equity gaps in ANC services utilization were observed in Ethiopia [35], and Kenya [36], and the quality-adjusted coverage was higher in UMICs than LMICs [113, 114]. In Brazil, the rate of the uptake of quality ANC was the least in the Northern province while the highest in the Southern province [77]. In Mexico, women had low-quality coverage if they were from Indigenous groups and living in remote regions [11, 115].

In many LMICs, uptake of 4ANC visits and interventions was the highest among socioeconomically advantaged populations [58, 68, 112]. The poorest groups had the highest rate of non-utilization of ANC visits in Pakistan and India [56, 75]. There were huge disparities in the quality of ANC in India’s Eastern and Central provinces [75].

### Effectiveness

The ANC quality was inversely associated with preterm birth and neonatal mortality [116, 117]. For example, newborns from women of the lowest quantiles of Indigenous in Mexico gained weight if they received 75% of the contents of ANC [115].

## Discussion

This study reviewed ANC quality using the Donabedian model of care. Several input, process, and output factors contributed to the optimal quality of ANC services. Input-related factors were poor readiness, lack of essential supplies and an inadequately trained workforce. Women of disadvantaged backgrounds and living in rural areas received poor-quality ANC care. Studies reported quality ANC is equated to an adequate number of ANC visits; however, other components of technical quality (skilled, timely, and adherence to protocols) have had little attention. Furthermore, the perceived quality of care, such as effective communication and client satisfaction, has received little attention in many LMICs. Studies reported low-quality adjusted coverage of ANC services and high equity gaps.

### Input

Several input factors (infrastructure, commodities, and health workforces) contributed to poor ANC quality in remote and rural areas of LMICs. Health facility readiness attributes are the foundation of the provision of quality ANC services [20, 119]. Structural facility readiness is essential quality ANC services that missed service provision at the service delivery point [35]. Evidence suggests better facility readiness can ensure better technical quality ANC services [31]. Potential supply-side strategies for input quality improving system inputs, provision of essential physical resources (medicine, equipment, and better infrastructure) and training health workforces [53]. Community-based healthcare workers’ recruitment, deployment and retention in rural and underprivileged areas improve their working conditions [120].

The current review showed several structural social determinants of health influenced the uptake of quality health services. Women of socially disadvantaged groups had received poor-quality ANC services. Although health system interventions cannot address such social disadvantages, targeted program approaches have potential to ensure quality ANC services. Identifying poor and marginalized communities (home visits, group ANC visits, and community models of care led by midwives) can improve ANC quality [31, 121].

Additionally, several modifiable characteristics of women influence the uptake of quality ANC services. Women empowerment and networking with women, improving local transportation systems, planned pregnancy at 25–30 years, and media exposure to pregnancy and childbirth were factors of uptake of quality ANC services. Non-health and health system interventions can modify several intermediary factors. For example, building local bridges and road networks can improve access to health facilities. In contrast, mobilization of local radio can disseminate health education and information on pregnancy-related issues [48]. Health system approaches also inform women to seek health care during pregnancy, including education on birth preparedness and complication readiness and health education session on pregnancy complications [68].

Moreover, some contextual approaches of service delivery (provision of ANC in groups, tasking shifting, and use of digital tools addressing demand and supply side constraints) improved the uptake of quality ANC. Implementation and scaling of such approaches require the resources and capacity of health systems. Closely monitoring ANC quality and delivery models, health workforce support, appropriate electronic technologies, integrated care, a woman-friendly perspective, and adequate infrastructure [122]. To design and implement innovative models of care, health systems need to repurpose and reorient themselves to push for governance and regulation by setting standards, producing guidelines, ensuring best practices, and strengthening the quality and cost-effectiveness of services [123].

### Process

The current review revealed that countries focused on the completion of 4ANC visits. However, other ANC quality components have gotten little attention in many LMICs. Evidence suggested that only completion of 4ANC visits does not guarantee the uptake of recommended ANC interventions [124]. The provision of optimum quality care depends on the health system readiness (e.g., health workforce trained on ANC). The availability of guidelines and standards is also vital to follow while in service delivery. Some health systems face a shortage of resources to ensure the facilities’ lab facilities. The provision of laboratory facilities (e.g., blood sugar tests) could identify gestational diabetes. In the first ANC visit, if healthcare providers all recommended interventions (e.g., adherence to the protocols), women could attend subsequent visits. The technical component of care (skilled, timely, adequate and adherence to guidelines) is vital for better health outcomes [31, 125]. Thus, delivering technical interventions through trained care providers is essential, especially in underserved communities. Health system efforts require targets to improve multiple dimensions of quality care [68].

The social quality or experience of care is an important component of the process of care. Respectful maternity care has received high attention in global policy and research. However, at the national level, there were several challenges in effective communication and poor client satisfaction in ANC services. System inputs need to focus on improving communication skills and attitudes to ensure social quality. Communication is the providers’ primary focus, and service users require to ask the providers questions (encouragement, awareness of the health care, empowerment to ask questions, and culturally safe care) [126]. The quality of ANC refers to care beyond coverage and attention to response-based services [43]. Thus, efforts require reframing to improve technical and social quality of ANC services, especially in low-income settings, to reach the unserved populations.

### Output

There has been an increase in the uptake of overall coverage of ANC visits. In the large body of literature, quality of care of ANC is equated to 4ANC visits. Quality of care is beyond adequate care (which is commonly used quality measurement approach at the population level); however, little research has been done on quality-adjusted coverage of ANC services. In addition,  there is limited information on ANC interventions at the user’s level that could be used to measure the quality-adjusted coverage of ANC at the population level. Additionally, little research has been done on quality-adjusted coverage in LMICs [68]. As current literature focuses on the number of contacts as the quality of care, there is a need to measure quality-adjusted coverage using population-level household survey data such as demographic and health surveys [124]. Quality-adjusted coverage can be improved by focusing on care processes, especially technical quality. Measuring the population-level coverage of quality health services and identifying women with poor access to quality ANC services are initial steps to address the equity gaps. These steps potentially improve access to quality antenatal services among most marginalized communities and regions where maternal and newborn mortalities are the highest.

This study synthesis evidence from the LMICs and identifies that disadvantaged populations have received suboptimal quality ANC services. In high-income countries, some disadvantaged groups (e.g., immigrants or indigenous groups) face several barriers to accessing health services. For instance, culturally and linguistically diverse groups in Australia experienced language and cultural barriers while accessing health services [127]. In addition, aboriginal women in northern Australia had poor adherence to local guidelines for follow-up of highly prevalent problems (e.g., anaemia, smoking, urinary tract infections and sexually transmitted infections) [98]. Similarly, in the United Kingdom, Black, Asian and Minority Ethnic (BAME) pregnant women faced several barriers and facilitators that prevented them from utilising the maternity services, warranting a provision for more culturally competent interventions [128]. Thus, pregnant women of these disadvantaged populations living in high-income countries must be prioritised for quality ANC services to ensure better pregnancy outcomes.

### Implications for program and research

This study provided insights into the multiple dimensions and factors contributing to the quality of ANC. This study synthesised several inputs and process factors of quality ANC. Current ANC quality research and programs focus on completing 4ANC visits but limited focus on structural inputs and the technical and social components of quality health care. Quality of care is complex and requires multiple dimensions of care. For example, respectful maternity care is important in gaining momentum, and it can be part of service delivery culture rather than processing by ticking the box only. High-income countries have implemented all components of quality, but still, some disadvantaged groups (imigrants) face barriers to receiving social quality. In LMICs, there are plenty of areas to improve the quality of ANC.

### Strengths and limitations

This study systematically reviewed the available evidence on the quality of ANC. The evidence was thematically synthesised and presented in an input-process-output model. The findings of this study could provide research, policy, and program insights to deliver health services for the improved quality of ANC. Ensuring the quality of ANC services is vital to realise universal health coverage and to achieve sustainable development goals. Nonetheless, the quality of ANC service depends on health system factors. Therefore, findings from this study could signal which factors to consider and where to focus on for system performance to deliver quality ANC. Identifying challenges could be the beginning steps toward further research agendas for access to health services for underserved communities and populations. We conducted a thematic synthesis that provides essential perspectives on the quality of ANC services in line with the research question; however, such analysis can miss the details of the country-specific findings and issues of a specific subsection of populations.

## Conclusion

In many LMICs, health facilities had poor readiness for quality ANC services, delivered poor technical quality care, and lacked effective communication and client satisfaction. Women of disadvantaged groups and from rural areas received poor-quality ANC services. Several health systems inputs (e.g., infrastructure, lack of trained workforce and supplies), and processes factors influenced quality of ANC. Inputs are linked with workforces, commodities, and individual factors that must be prioritised to improve the technical and perceived quality. Several approaches service of delivery (e.g., group ANC visit, and early ANC first visit) could improve access to high quality ANC service. Health systems need to focus on technical quality (skilled, timely, adequate, and adherence to guidelines) and social quality (effective communication and improved client satisfaction). Availability of standards and guidelines, training of health workers, technical support visits, and mentoring of staff could improve providers’ technical and social skills for delivering high-quality care. Household survey data can be used to estimate the quality-adjusted coverage of ANC services at the population level. Identifying coverage of quality ANC service is vital among the most disadvantaged populations. Designing and implementing targeted program approaches can reduce equity gaps in accessing quality ANC services.

## Supplementary Information

**Additional file 1: Appendix Table S1.** Preferred Reporting Items for Systematic reviews and Meta-Analyses extension for Scoping Reviews (PRISMA-ScR) Checklist. **Appendix Table S2.** Search strategy. **Appendix Table S3.** Data extracts of quality of ANC services.

## Data Availability

All data generated or analysed during this study are included in this published article [and its supplementary information files].

## Abbreviations

ANC: Antenatal Care
PRISMA-ScR: Systematic Reviews and Meta-Analyses Extension for Scoping Reviews
LMICs: Low and Lower- or Middle-Income Countries
SSA: Sub-Saharan Africa
SA: South Asia
MMR: Maternal Mortality Ratio
NMR: Neonatal Mortality Rate
MNH: Maternal and Newborn Health
WHO: World Health Organisation
SBAs: Skilled Birth Attendants
SES: Socioeconomic Status
