# Supplementary material for: Input, process, and output factors contributing to quality of antenatal care services: a scoping review of evidence
Source: BMC Pregnancy Childbirth. 2022 Dec 28;22:977. doi: 10.1186/s12884-022-05331-5 (PMC9795647; doi:10.1186/s12884-022-05331-5)
Supplement: Supplementary file 1 — Additional file 1: Appendix Table S1. Preferred Reporting Items for Systematic reviews and Meta-Analyses extension for Scoping Reviews (PRISMA-ScR) Checklist. Appendix Table S2. Search strategy. Appendix Table S3. Data extracts of quality of ANC services. [file 12884_2022_5331_MOESM1_ESM.pdf]

**Appendix Table S1: Preferred Reporting Items for Systematic reviews and Meta-Analyses extension for Scoping Reviews (PRISMA-ScR) Checklist**

| SECTION | ITEM | PRISMA-ScR CHECKLIST ITEM | Page, line # |
|---|---|---|---|
| TITLE | | | |
| Title | 1 | Identify the report as a scoping review. | 1-2 |
| ABSTRACT | | | |
| Structured summary | 2 | Provide a structured summary that includes (as applicable): background, objectives, eligibility criteria, sources of evidence, charting methods, results, and conclusions that relate to the review questions and objectives. | 17-50 |
| INTRODUCTION | | | |
| Rationale | 3 | Describe the rationale for the review in the context of what is already known. Explain why the review questions/objectives lend themselves to a scoping review approach. | 97-103 |
| Objectives | 4 | Provide an explicit statement of the questions and objectives being addressed with reference to their key elements (e.g., population or participants, concepts, and context) or other relevant key elements used to conceptualize the review questions and/or objectives. | 1-4=105 |
| METHODS | | | |
| Protocol and registration | 5 | Indicate whether a review protocol exists; state if and where it can be accessed (e.g., a Web address); and if available, provide registration information, including the registration number. | NA |
| Eligibility criteria | 6 | Specify characteristics of the sources of evidence used as eligibility criteria (e.g., years considered, language, and publication status), and provide a rationale. | 121-132 |
| Information sources* | 7 | Describe all information sources in the search (e.g., databases with dates of coverage and contact with authors to identify additional sources), as well as the date the most recent search was executed. | 113-119 |
| Search | 8 | Present the full electronic search strategy for at least 1 database, including any limits used, such that it could be repeated. | 113-119 |
| Selection of sources of evidence† | 9 | State the process for selecting sources of evidence (i.e., screening and eligibility) included in the scoping review. | 133-148 |
| Data charting process‡ | 10 | Describe the methods of charting data from the included sources of evidence (e.g., calibrated forms or forms that have been tested by the team before their use, and whether data charting was done independently or in duplicate) and any processes for obtaining and confirming data from investigators. | 133-156 |
| Data items | 11 | List and define all variables for which data were sought and any assumptions and simplifications made. | 149-154 |
| Critical appraisal of individual sources of evidence§ | 12 | If done, provide a rationale for conducting a critical appraisal of included sources of evidence; describe the methods used and how this information was used in any data synthesis (if appropriate). | 126-132 |
| Synthesis of results | 13 | Describe the methods of handling and summarizing the data that were charted. | 146-156 |
| RESULTS | | | |

| SECTION | ITEM | PRISMA-ScR CHECKLIST ITEM | Page, line # |
|---|---|---|---|
| Selection of sources of evidence | 14 | Give numbers of sources of evidence screened, assessed for eligibility, and included in the review, with reasons for exclusions at each stage, ideally using a flow diagram. | 157-177 |
| Characteristics of sources of evidence | 15 | For each source of evidence, present characteristics for which data were charted and provide the citations. | 157-172 |
| Critical appraisal within sources of evidence | 16 | If done, present data on critical appraisal of included sources of evidence (see item 12). | NA |
| Results of individual sources of evidence | 17 | For each included source of evidence, present the relevant data that were charted that relate to the review questions and objectives. | Appendix |
| Synthesis of results | 18 | Summarize and/or present the charting results as they relate to the review questions and objectives. | Page 10 1-249 |
| DISCUSSION | | | |
| Summary of evidence | 19 | Summarize the main results (including an overview of concepts, themes, and types of evidence available), link to the review questions and objectives, and consider the relevance to key groups. | Page 17 246-256 |
| Limitations | 20 | Discuss the limitations of the scoping review process. | 352-366 |
| Conclusions | 21 | Provide a general interpretation of the results with respect to the review questions and objectives, as well as potential implications and/or next steps. | 376-384 |
| FUNDING | | | |
| Funding | 22 | Describe sources of funding for the included sources of evidence, as well as sources of funding for the scoping review. Describe the role of the funders of the scoping review. | 396 |

**Appendix Table S2: Search strategy**

| Database | Keywords |
|---|---|
| PubMed | A: ("Antenatal care"[Title/Abstract] OR "antenatal service*"[Title/Abstract] OR "prenatal care"[Title/Abstract] OR "prenatal service*"OR "pregnancy care"[Title/Abstract] OR "perinatal care"[Title/Abstract] OR "perinatal service*"[Title/Abstract] OR "antenatal check-up*"[Title/Abstract] OR "antenatal visit*"[Title/Abstract] OR "antenatal follow-up"[Title/Abstract] OR "antenatal follow up"[Title/Abstract])<br>B: ("Quality of care"[Title/Abstract] OR quality[Title/Abstract] OR accessibil*[Title/Abstract] OR acceptabilit*[Title/Abstract] OR "skilled care"[Title/Abstract] OR affordab*[Title/Abstract] OR effective*[Title/Abstract] OR efficien*[Title/Abstract] OR frequen*[Title/Abstract] OR continu*[Title/Abstract] OR adequa*[Title/Abstract] OR time*[Title/Abstract] OR "antenatal care intervention*"[Title/Abstract] OR coverage[Title/Abstract] OR utiliz*[Title/Abstract] OR "respect* care"[Title/Abstract] OR "dignified care"[Title/Abstract] OR satisf*[Title/Abstract] OR safe*[Title/Abstract] OR "women-centered"[Title/Abstract] OR "patient-centered"[Title/Abstract] OR equit*[Title/Abstract] OR cost*[Title/Abstract])<br>C: A AND B |

| | |
|---|---|
| Embase | A: ('antenatal care': ab OR 'antenatal service*': ab OR 'prenatal care':ab OR 'prenatal service*':ab OR 'pregnancy care':ab OR 'perinatal care':ab OR 'perinatal service*':ab OR 'antenatal check-up*':ab OR 'antenatal visit*':ab OR 'antenatal follow-up':ab OR 'antenatal follow up':ab)<br>B: ( 'quality of care':ab OR quality:ab OR accessibil*:ab OR acceptabilit*:ab OR 'skilled care':ab OR affordab*:ab OR effective*:ab OR efficien*:ab OR frequen*:ab OR continu*:ab OR adequa*:ab OR time*:ab OR 'antenatal care intervention*':ab OR coverage OR utiliz*:ab OR 'respect*:ab care':ab OR 'dignified care':ab OR satisf*:ab OR safe*:ab OR 'women-centered':ab OR 'patient-centered':ab OR equit*:ab OR cost*:ab)<br>C: A AND B |
| Scopus | A: ("Quality of care" OR quality OR accessibil* OR acceptabilit* OR "skilled care" OR affordab* OR effective* OR efficien* OR frequen* OR continu* OR adequa* OR time* OR "antenatal care intervention*" OR coverage OR utiliz* OR "respect* care" OR "dignified care" OR satisf* OR safe* OR "women-centered" OR "patient-centered" OR equit* OR cost* )<br>B: ("Antenatal care" OR "antenatal service*" OR "prenatal care" OR "prenatal service*" OR "pregnancy care" OR "perinatal care" OR "perinatal service*" OR "antenatal check-up*" OR "antenatal visit*" OR "antenatal follow-up" OR "antenatal follow up" )<br>C: A AND B |
| Google scholar | A: Antenatal care, antenatal service, prenatal care, prenatal service, pregnancy care, perinatal care, perinatal service, antenatal check-up, antenatal visit, antenatal follow-up, antenatal follow up,<br><br>B: Quality of care, quality, accessibility, acceptability, skilled care, affordability, effective, efficiency, frequency, continuity, adequacy, time, antenatal care intervention, coverage, utilization, respectful care, dignified care, satisfaction, safe, women-centered, patient-centered, equity, cost |

**Appendix Table S3: Data extracts of quality of ANC services**

| Study | Country | Study | Aim | Quality of ANC | Key findings |
|---|---|---|---|---|---|
| Magoma 2011 [1]. | Tanzania | Qualitative | To document the time spent by health care providers on ANC | Timely care Adequate care | The time spent on each component of care was also greater among in observed for subsequent ANC consultations. Birth plans, counselling on HIV were discussed but danger signs during pregnancy) were rarely discussed |
| Conrad et al 2012 [2] | Uganda | mixed methods | To appraise the quality of ANC services in a rural district of Uganda | Bottlenecks of poor quality | bottlenecks of service delivery e.g., ineffective organization of educational sessions; selective omission of certain services; lack of explanation of clinical and lab procedures, failure to link the procedures with preventive information; and lack of respect for clients. |

| Study | Country | Study | Aim | Quality of ANC | Key findings |
|---|---|---|---|---|---|
| Kumban et al 2012 [3] | Malawi | Qualitative | To describe women's perceptions of perinatal care among the women delivered at a district hospital | Enablers of good care Barriers of good care | Good care if there was respect, confidentiality, privacy, and normal delivery. Providers' attitudes, delay in providing care, inadequate care, and unavailability of delivery attendants were subthemes under unsatisfactory care. |
| Kyei et al 2012 [4] | Zambia | Quantitative | To the level of ANC service provision at health facilities in Zambia on a national scale and compared this to the quality of ANC received by expectant mother | Poor readiness for ANC services High discontinuation and poor-quality care | Only 45 antenatal facilities (3%) fulfilled criteria for optimum ANC service, 47% of facilities provided adequate service, 94% of mothers reported at least one ANC visit with a skilled health worker and 60% attended at least four visits, only 29% of mothers received good quality ANC, and only 8% of mothers received good quality ANC and attended in the first trimester. |
| Naariyong et al [5]. | Ghana | Quantitative | To compare the quality of ANC between Community-based Health Planning and Services | CHPS improved the better ANC care | Participants in the CHPS areas had full utilization score, received HIV testing, and anti-malarial prophylaxis during the ANC period, scoring high on the index of knowledge was not significant with the CHPS exposure |
| Nyamtema et al 2012 [6]. | Tanzania | Quantitative | To identify the factors of quality of care of ANC | Knowledge is adequate for good care | Fifty-two (20%) severe maternal morbidities were attributed to substandard ANC. Of these 39 had severe anaemia and eclampsia combined. Substandard ANC was due to a shortage of staff, equipment, and consumables. |
| Tetui et al 2012 [7]. | Uganda | Qualitative | To assess the quality of ANC | Inadequate system inputs Inadequate care | There was an overall staffing gap of over 40%, while infection control facilities, drugs and supplies were inadequate. There was good existence of infrastructure and equipment for ANC services. Counselling for risk factors and birth preparedness was poorly done; essential tests were not done for most clients. |

| Study | Country | Study | Aim | Quality of ANC | Key findings |
|---|---|---|---|---|---|
| Atinga et al 2012 [8] | Ghana | Quantitative | To achieve MDG5 through implantation of free maternal health service system | Age of mothers and education predicts ANC quality. | Received good care if aged 30 and 34 women, women with junior/senior high education reported ANC quality as good. Distance to the health facilities proximity of HFs, factors (pleasant interaction with providers, privacy during consultation, attentiveness of providers, adequate facilities and availability of drugs) ANC quality. |
| Ejigu et al 2013 [9]. | Ethiopia. | Quantitative | To assess the quality of ANC services at public health facilities. | poor quality care especially lab and supplies. Lack of reagents | VDRL test, blood group and Rhesus factor tests were done only for 73 (19.8%) and 133 (36.0%) of the women, 236 (64.0%) of the mothers missed the opportunity to receive iron/folic acid supplements during their ANC visit. Only 226 (61.2%) women had their conjunctiva checked for anaemia. Lack of reagents partly explained the problems observed in recommending care components. |
| Manithip et al 2013 [10]. | Lao PDR | Qualitative | To explore the health-care providers' performance and their own perceptions | Health workforce factors. Poor readiness in lower-level facilities | Poor quality and performance of ANC services in rural HFS, lack of routines, scarce or insufficient equipment and limited skills among providers. HWs factors- competence and motivation to work with ANC, lack of patient feedback and expressed a need for support from health-care superiors, less well-equipped and supplied, heavier workload, short consultation time (5 mins). |
| Bar-Zeev et al 2014 [11] | Australia | mixed method | To assess adherence to antenatal guidelines by clinicians | Adherence with guideline | Women attended ANC, high adherence to some routine ANC screening guidelines. But poor adherence to local guidelines for follow-up of highly prevalent problems (e.g., anaemia, smoking, UTIs, and STIs) |
| Duysburgh et al., 2014 [12] | Ghana | Qualitative | To improve MNH by improving the quality of antenatal and childbirth care at rural HFs. | Poor quality care<br><br>Critical gaps of ANC | Satisfactory quality of routine ANC. Counselling and health education practices needs improvement; laboratory investigations are often not performed; examination and monitoring of mother and newborn during childbirth are inadequate; partographs are often not used and poorly completed; and equipment to provide assisted vaginal deliveries was absent. |
| El Aty et al 2014 [13] | Oman | Quantitative | To the adequacy of ANC utilization of Omani ever-married women | Poor attendance in first trimester Poor adequacy of care | Women who had 4ANC visits, attended ANC in the 1st trimester and received care from trained personnel were 96.8%, 74.9% and 99.1%, The adequacy of ANC (use and sufficiency of recommended basic services) for the surveyed women was 53.8%. Being pregnant with the 1st baby was predictor of adequacy of ANC. |
| Hodgins et al 2014 [14] | Multicountry | Quantitative | To analyse specific elements of care received among | Low population effective coverage. Low coverage of | Coverage for specific elements of care among women reporting 4+ ANC visits was low for most of the specific elements assessed. BP and TT performed best, with median quality–coverage gaps of 5% and 18%. |

| Study | Country | Study | Aim | Quality of ANC | Key findings |
|---|---|---|---|---|---|
| | | | women reporting 4 or more ANC visits. | adequate care. Low 4ANC visits | High gaps - iron–folate supplementation (72%) and malaria prevention (86%). Low uptake of (referring population EC) than among only those who had received ANC 4+ visits. Countries had lower average coverage for care content than ANC 4+. Half the women had 4+ANC visits and 85% had at least one visit. Health education, iron supplementation, blood pressure measurement and tetanus toxoid were ANCs more commonly received components. |
| Joshi et al 2014 [15] | Nepal | Quantitative | To identify factors associated with 1) attendance at four or more ANC visits and 2) receipt of good quality ANC. | Factors of predictor of good quality | Older age, higher parity, and higher levels of education and household economic status of the women were predictors of both attendances at 4+ visits and receipt of good quality ANC. Women who did not smoke, had a say in decision-making, whose husbands had higher levels of education and were involved in occupations other than agriculture, receiving their ANC from a skilled provider, in a hospital, living in an urban area and being exposed to general media. |
| Majrooh et al 2014 [16]. | Punjab | Mixed methods | To assess the coverage and quality antenatal care in the primary health care facilities in 'Punjab' province of 'Pakistan'. | High discontinuation in the subsequent visits | Enrolment for ANC was 55.9% and dropout was 32.9% in subsequent visits. The quality of services regarding assessment, treatment and counselling was extremely poor. The reasons for low coverage and quality were the distant location of facilities, deficiency of facility resources, indifferent attitude, and non-availability of the staff. Lack of client awareness about importance of ANC and self-empowerment for decision-making to seek care were also responsible for low coverage. |
| (Saizonou et al. 2014 [17] | Benin | Qualitative | To assess the performance of antenatal services | Satisfactory health system inputs | The health system inputs for ANC service the inputs, procedures, and satisfaction of the pregnant women, was satisfactory. The services organization, health care environment, patient reception and interpersonal communication were the elements which showed deficiencies. |
| Achia et al 2015 [18] | Kenya | Quantitative | To examine individual and community level factors associated with adequate use of maternal antenatal health services. | Factors of adequacy of care | The individual-level factors associated with adequate use of MHC- the greater educational attainment by the woman or her partner, higher socioeconomic status, access to medical insurance coverage, and greater media exposure. Greater community ethnic diversity, higher community-level socioeconomic status, and greater community-level health facility deliveries were the contextual-level factors associated with adequate use of MHC. |

| Study | Country | Study | Aim | Quality of ANC | Key findings |
| --- | --- | --- | --- | --- | --- |
| Fagbamigbe et al 2015 [19] | Nigeria | Quantitative | To assess the quality of ANC services in Nigeria | Low uptake of interventions in routine visits | IPD and IPT were given to only 20.7 % and 37.6 % respectively in Nigeria. 41.7 % had HIV test and obtained results. Only 4.6 % of women received good quality of ANC while nearly 1.0 % did not receive any of the components. About 11.3 % of the attendees had minimum acceptable quality of ANC. Quality was higher among users who initiated ANC early, with 4 ANC visits, attended to by skilled health workers, attended government and private hospitals and clinics, who lives in urban areas, having higher educational attainment, upper wealth quintiles and attended to by skilled ANC provider. |
| Kambala et al 2015 [20] | Malawi | Quantitative | To measure women's perceived quality of maternal and newborn care using a composite scale | Perceived quality | A high perceived quality of care - interpersonal relations, conditions of the examination rooms and nursing care services. Self-introduction by the health worker, explanation of examination procedures, consent seeking, encouragement to ask questions, confidentiality protection and being offered to have a guardian during delivery were associated with a high-quality rating of interpersonal relations for antenatal and delivery care services, literate, never experienced a still birth and, first ANC visit were associated with a high-quality rating of room conditions for ANC service. |
| Villadsen et al 2015 [21] | Ethiopia | Qualitative | To design a participatory ANC strengthening intervention and assess effectiveness on quality of ANC. | Individual and organisation factors of quality | The continued attention to the ANC provision during implementation stimulated increased priority of ANC among health care providers. The organizational structure of the facilities and lack of continuity in care provision turned out to be a major challenge for implementation. positive effect of the intervention on health education on danger signs during pregnancy, laboratory testing for blood tests other than HIV, health problem identification, and satisfaction with the service. Maternal education impacts outcomes significantly. |
| Bayou et al 2016 [22] | Ethiopia | Quantitative | To report on the findings of antenatal care adequacy among slum residents | Inadequate quality among poor better quality in private facilities | inadequate antenatal care services among slum resident women-initiated ANC early, received adequate care contents and had overall adequate care services. Educational status and place of ANC visits are determinant factors for adequacy of ANC. Women with secondary and above educational status received adequate care. Clients of private healthcare facilities received adequate ANC. |
| Cohen et al 2016 [23] | Kenya | Qualitative | To analyse the ANC quality | Private health services | Active patients are increasingly likely to pay for private care and receive a higher quality of care over their pregnancy. |

| Study | Country | Study | Aim | Quality of ANC | Key findings |
|---|---|---|---|---|---|
| Heredia-Pi et al 2016 [24] | Mexico | Quantitative | To propose an ANC classification for measuring the COC | Two third received adequate care | Most women received antenatal care during their last pregnancy, only 71.5% received maternal health care classified as adequate with geographical inequity.<br>The probability of receiving adequate ANC was higher among women of higher socioeconomic status, with more years of schooling and with health insurance |
| Servan-Mori et al 2016.[25] | Mexico | Quantitative | To examine the role of components of adequate ANC in disparities in birth | Indigenous women poor quality | Coverage of adequate ANC (timely, frequent, and complete care) is lower among indigenous (59%,) than non-indigenous (68%) women. Among indigenous women, greater newborn weight gains are achieved in the lowest quantiles if they have access to 75% of the content of ANC compared with those that did not have access. Complete ANC appears to differentially affect indigenous women at the bottom of the birth weight distribution. |
| Do et al 2017 [26] | Namibia | Quantitative | To assess the quality of ANC, an entry point to the health system for many women | Long waiting time and poor client satisfaction | Long waiting time was a common problem and was generally more serious in hospitals and health centers than in clinics and smaller facilities; it was consistently associated with lower client satisfaction. the provider's technical preparedness may not be sufficient to provide good-quality services and to ensure client satisfaction. |
| Fagbamigbe et al. [27] | Nigeria | Quantitative | To assess trend of the timings of first ANC visit | Late initiation of ANC | Prevalence of ANC use was 60.5% in 2008 and 65.8% in both 2003 and 2013. Less than one-third (32.3%) of the women accessed ANC within first 3 months of pregnancy, with highest rate (41.7%) among women with higher education and those from North Central Nigeria (42.7%). |
| Koroma et al. 2017 [28]. | Northern Sierra Leone | Quantitative | To examine the quality of free antenatal services | Poor quality ANC | The quality of services was poor. Only 27% of women were examined, 2% were screened on their first ANC visit and 47% received interventions. |
| Leslie et al [29] | SSA | Quantitative | to examine effective coverage of maternal and child health services. | Poor quality ANC services | Quality of care was poor, with few regions demonstrating more than 60% average performance of basic clinical practices in any service. Effective coverage across all eight countries averaged 28% for antenatal care. Coverage and quality were not strongly correlated at the subnational level; effective coverage varied by as much as 20% between regions within a country. |
| Leslie et al 2017 [30] | Haiti, and SSA | Quantitative | To assess whether the most measured attribute of health facilities in LMICs | Low adherence to guideline | Adherence to evidence-based guidelines was low, with an average of 61% in ANC. Correlation between infrastructure and evidence-based care was low. Facilities with similar infrastructure scores delivered care of widely varying quality in each service. |

| Study | Country | Study | Aim | Quality of ANC | Key findings |
|---|---|---|---|---|---|
| Miltenburg et al [31] | Tanzania | Mixed methods | To assess the quality of antenatal care provision from a holistic perspective | Insufficient resource for quality care | Variable performance of routine ANC services, explained by insufficient resources. Poor performance was also observed for appropriate history taking, attention for client's wellbeing, basic physical examination and adequate counselling and education. |
| Muchie et al 2017 [32] | Ethiopia | Quantitative | To examine individual, household, and community level factors | Low recommended and timely visits | Only 33.0% of women completed the recommended visits. Of them 56.5% had at least one ANC visit. 37.4% visited in their first trimester. Completing the recommended visits was negatively associated with women with lower educational levels, lower economic conditions, higher birth order, and rural residence. |
| Serván-Mori et al 2017 [33] | Mexico | Quantitative | To assess maternal health metrics and system performance. | Low quality ANC | comprehensive access to ANC declines steeply in both groups as we move along the maternal healthcare continuum. Access to comprehensive antenatal care is far from optimal. |
| Sharma et al 2017 [34] | Kenya | Quantitative | To assess whether high quality maternal care | Poor clinical quality of ANC | Clinical quality of ANC was 0.52 out of 1 respectively, compared to 0.68 for structural inputs to care. Maternal healthcare quality varied by poverty level. Population access to a minimum standard (0.75 of 1.00) of quality maternal care was both low and inequitable: only 17% of all women. |
| Solnes et al 2017 [35] | Tanzania | Mixed methods | To assess the quality of antenatal care provision from a holistic perspective | Poor resources and poor quality | Performance of routine ANC services, partly explained by insufficient resources. Poor performance was also observed for appropriate history taking, attention for client's wellbeing, basic physical examination and adequate counselling and education. |
| Ataguba et al 2018 [36] | SSA | Quantitative | To apply a novel index of service coverage using ANC | Poor ANC as per protocol | Dsparities exist in country ranking as some countries, e.g., Cameroon, Benin Republic and Nigeria are ranked better using the ANC4+ indicator but poorly using the proposed index. |
| Benova et al [37] | 10 LMICs | Quantitative | To describe ANC coverage and operationalised indicators. | Poor adherence to protocol | Receipt of the six routine components varied widely; blood pressure measurement was the most reported, and urine test and information on complications were the least. Among the subset of women starting ANC in the first trimester and receiving 4+ visits, the percentage receiving all six routinely measured ANC components was low, ranging from 10% (Jordan) to around 50% in Nigeria, Nepal, Colombia and Haiti. |

| Study | Country | Study | Aim | Quality of ANC | Key findings |
|---|---|---|---|---|---|
| Coley et al 2018 [38] | USA | Qualitative | To compare perceptions of prenatal care quality between African American and mixed-race mothers | Tailoring and culturally sensitive care improve quality | Mothers and providers valued the tailoring of care based on individual needs and functional patient-provider relationships as key elements of prenatal care quality. Providers acknowledged the need for knowing patients' social context, but mothers and providers differed in perspectives of “culturally sensitive” prenatal care. Although most mothers had positive prenatal care experiences, mothers also recalled multiple complications with providers’ negative assumptions and disregard for mothers’ options in care. |
| Ejigu et al 2018 [39] | Ethiopia | Quantitative | To assess the linkage between ANC quality among pregnant women | Good ANC quality higher institutional delivery | Only about one-third received acceptable quality of ANC services. In one health facility syphilis test was not done at all for the last two years. The odds of giving birth at health institution among pregnant women who received acceptable ANC quality service was 3 times higher. |
| . Katemba et al 2018 [40] | Zambia | Quantitative | To assess the quality of ANC services | Low ANC quality, especially lab services | 47.1% of pregnant women received high-quality ANC. Six key ANC interventions were considered, among which urine (36.7%) and blood (46.8%) testing were the least received basic components of ANC. Women with secondary education had higher odds of receiving high-quality ANC. Women staying with their husband/partners had lesser odds of receiving high quality ANC; |
| Rurangirwa et al 2018 [41] | Rwanda | Quantitative | To investigate the ANC providers' current practices. | Midwife provided services | Midwives did somewhat better than nurses in reporting these conditions. midwives informed pregnant women about pregnancy-related issues. |
| Yeoh et al 2018 [42] | Malaysia | Quantitative | To assess the adequacy of ANC and its association with pregnancy outcomes | Good quality ANC reduce preterm birth | Sixty-three percent of women showed higher than recommended ANC utilization; 52% had <80% of recommended routine care content. Preterm births were lower among women with adequate level of utilization compared with inadequate and intensive levels. women who received inadequate care content were associated with a higher prevalence of preterm birth. |
| Afulani et al 2019 [43] | Kenya | Quantitative | To assess the quality of ANC women received in Migori county, | Gaps in communication with poor satisfaction | Key gaps demonstrated in communication. About half of women were not educated on pregnancy complications. about one-third did not often understand the purposes of tests and medicines received and did not feel able to ask the health care provider questions. women who were literate, employed, and who received all their ANC in a health center had higher experiences scores. |

| Study | Country | Study | Aim | Quality of ANC | Key findings |
|---|---|---|---|---|---|
| Alyahya et al 2019 [44] | Jordan | Qualitative | To explore the quality of ANC in Jordan | Better communication improved quality | Reasons for this included longer consultation time, a higher quality of services, better interpersonal and communication skills of healthcare providers, better treatment, more advanced equipment and devices, availability of female obstetricians, and more flexible appointment times. These women only perceived public hospital services as necessary in pregnancy-related complications and labour, as the costs of private sector services in such cases are too high. |
| Jo et al [45] | Bangladesh | Qualitative | To determine service subcomponents and provider and patient costs of ANC services | Low services complaints in physical exam and test | High compliance (> 50%) of service subcomponents were observed in blood pressure monitoring, weight measurement, iron and folate supplementation given, and tetanus vaccine, while lower compliance of service subcomponents (< 50%) were observed in some physical examinations such as oedema and ultrasonogram and routine tests such as blood test and urine test. |
| Mario et al [46] | Brazil | Quantitative | To assess the adequacy of prenatal care associated factors | Low quality care and test | Adequate prenatal care was more frequent among white women who performed prenatal care in the private health sector. The northern region had the lowest rate of adequate prenatal care, while the southeast region showed the highest rates. |
| Naz 2019 [47] | Pakistan | Mixed methods | To address socioeconomic inequalities of ANC. | Inequity in quality care uptake | a concentration of non-utilization of ANC amongst the poorest. The socioeconomic status contributed the largest percentage, 44%, followed by place of residence, at 21%, a husband's education at 14%, and the woman's age at 13%. |
| Okawa et al [48] | Myanmar | Quantitative | To assess adequate contact made by women and newborns with skilled care providers | Low adequate contact. Handbook was positively associated | The percentage of crude adequate contact was 60.9% for ANC. The percentage of quality-adjusted contact was 14.6% for ANC, Adequate contact was associated with receiving high-quality care at ANC, Being a teenager, low educational level, multiparity and low level in the household wealth index were negatively associated with adequate contact with healthcare providers for ANC. Receiving a maternal and child health handbook was positively associated with adequate contact for ANC and receiving high-quality ANC. |
| Owili et al 2019 [49] | SSA | Quantitative | To explore the determinants of quality of ANC | General readiness improves quality | Those with ANC guideline and central electricity supply were more likely to provide optimal information. Provider's qualification and experience were also important in information provision and clinical care independently. |

| Study | Country | Study | Aim | Quality of ANC | Key findings |
|---|---|---|---|---|---|
| Phommachanh et al. 2019 [50] | Lao PDR (Laos | Qualitative | To gain insight into the perceptions of stakeholders on both supply and demand sides of public ANC | Demand and supply side factors improve quality | The supply side included facilities, human resources, privacy and confidentiality, providers' behavior, attitudes, and ineffective communication skills. Demand side issues of providers' behavior, attitude, communication and unequal treatment, and the lack of privacy. Suggested solutions are training, effective materials, rewarding good role models, and building a feedback system. |
| Pugliese-Garcia et al [51] | Egypt | Quantitative | To describe the changes over time (1991-2014) in the use of ANC focusing on sector of provision | Low completion of ANC visits and interventions | 44% of women using public ANC reported eight or more visits compared to 71% in private ANC. 24% of ANC users received all seven care components with 10% of women reporting fewer than four ANC visits to 29% of women reporting eight or more. The poorest ANC users received all seven measured care components less often than the wealthiest. |
| Rios-Zertuche et al 2019 [52] | Multi-country of Latin America | Quantitative | To measure standardized, replicable matrix of quality | Low level of quality care | Routine interventions, such as quality ANC, immediate neonatal care, and postpartum contraception, had low levels of compliance. Records that complied with quality ANC ranged from 68.8% in Costa Rica to 5.7% in Guatemala. |
| Singh et al 2019 [53] | India | Quantitative | To assess the coverage and adequacy of ANC services available to women and associated factors | Inadequate ANC and disparity | A considerable proportion of women do not receive adequate ANC in India. Stark socio-economic and regional disparity was evident about the availability and provision of the quality of ANC among women. Women belonging to the poorest wealth quintile or the adolescent age group, illiterate women and those residing in the central and eastern regions of the country did not receive adequate ANC. About 70% of pregnant women received appropriate ANC, which was timely, delivered by skilled professionals, and sufficient in the number of visits made. |
| Thida et al [54] | Myanmar | Quantitative | To identify the types of HWs for ANC | Low perceived quality | Low perceived good quality ANC and delivery care for HDP and PPH. More than 85% of the participants rated quality of care for managing complications as good. |
| Yakob et al [55] | Ethiopia | Quantitative | To assess the quality and effective coverage of ANC services. | Poor effective coverage of ANC visit | The crude coverage 62% for ANC in Ethiopia in 2016. ANC quality was 34% and 81% received <50% of the recommended ANC clinical actions. When adjusting the crude coverage by the service quality, the mean EC of ANC services was 22%. |
| Adhikari et al 2020 [56] | Nepal | Quantitative | To analyse the sociodemographic correlates of the frequency and quality of ANC. | Poor quality care, with advantaged women received good quality | While 70% of the Nepalese women surveyed had at least four ANC visits, only 21% of these women received good-quality ANC. educated women and the women of rich wealth index were more likely to receive more antenatal visits. women living in rural areas, and those who had more than two children were less likely to receive a higher number of |

| Study | Country | Study | Aim | Quality of ANC | Key findings |
|---|---|---|---|---|---|
| | | | | | antenatal visits. Regarding the quality of ANC, educated husbands, rich wealth index and who had intended pregnancy, were more likely to receive good-quality ANC. |
| Albert et al 2020 [57] | Pakistan | Quanti tative | To assess the structural and procedural quality of ANC services | Inputs and communication for quality | The overall quality of ANC was rated as good (61%). A need to cultivate quality of care at public health facilities, train health workers in communication skills, and build technical capacity by continuing education and supportive supervision to train health-care providers to follow standard protocols for provision of quality ANC. |
| de Andrade et al 2020 [58] | Brazil | Quanti tative | To evaluate adherence for prenatal care by comparing first-time | Adolescent received poor prenatal care | adolescent group had lower attainment of almost all recommended components of prenatal care, with statistically significant differences for 2 blood glucose tests 2 VDRL tests, 2 Hb/Ht exams, and at least 6 consultations. |
| Benski et al 2020 [59] | Madaga scar | Quanti tative | To use mHealth to provide ANC to 1446 pregnant women in a rural area | Earlier ANC visit improved visit in second trimester | Women started to come earlier for their first ANC visit; more women attended their first ANC visit in the second trimester of pregnancy in 2019 than in the previous years, including age, educational level, gravidity, parity, infectious diseases, and level of anaemia. |
| Berehe et al 2020 [60] | Ethiopia | Quanti tative | To determine the quality of ANC | Suboptimal quality of care | The magnitude of good quality of ANC service was low. The frequently identified problems were inability to take full history, lack of proper counselling, poor healthcare provider and client interaction, improper registration, and a variation in providing quality of care in each visit. Quality of ANC was associated with residence, educational status gravidity, parity, and visit. |
| Dadras et al 2020 [61] | Iran | Quanti tative | To explore the sociodemographic factors and potential barriers associated with adequate ANC | Only one third of Afghan women received adequate quality | Almost a third of Afghan women in this study had adequate ANC (≥ 8 visits). The women in older age group, those with higher education and family income, women with longer length of stay, those of legal status had adequate ANC. the poor knowledge and attitude toward ANC, the poor quality of services; and the difficulties in access were the main obstacles toward adequate ANC. |
| Defar et al 2020 [62] | Ethiopia | Quanti tative | To the structural quality of ANC service provision in Ethiopian health facilities | Poor availability of tracer ANC items | The availability of specific services was very low. Tetanus toxoid. The mean availability among the ten tracer items (vaccination, folic acid, iron supplementation, and monitoring of HTP) for quality ANC services was 50%. Health canters, health posts and clinics scored lower ANC service readiness. The overall readiness index score was lower for private health facilities. Regional inequity was also observed. |

| Study | Country | Study | Aim | Quality of ANC | Key findings |
|---|---|---|---|---|---|
| Fauziah et al 2020 [63] | Indonesia | Quantitative | To assess the quality of ANC in rural and urban PHC | Poor optimal quality | The results indicate that 52.6% of ANC quality is categorized bad. Furthermore, there is different ANC quality based on body weight, the height of fundus uteri, and administration of iron tablet. |
| Harsha Bangura et al 2020 [64] | Nepal | Mixed methods | To improve utilization, quality of care, and the patient experience. | Group ANC approach was effective | Group ANC approach- 85.7% of visits completing all process elements, and high content fidelity, with all village clusters meeting the minimum target frequency for 80% of topics. Group gestational matched and stable group intervention feasible through training, documentation, feedback, and logistics. group ANC provided in collaboration with local government clinics has the potential to provide accessible and high-quality ANC. |
| Hategeka et al 2020 [65] | Rwanda | Quantitative | To assess effective coverage and equity of MCH services in MDG era | Low effective coverage of ANC services | In 2015, average effective coverage was 33.2% across all five MCH services, 30.1% for ANC with contrast to contact coverage 67.3%. There as increased EC, which was associated with widening socioeconomic inequalities. |
| Merrell [66] | Guinea | Quantitative | To explore the relationship between women's empowerment and quality ANC care. | Women empowerment improved quality | women's empowerment (decision-making, literacy/access to magazines, monogamous relationship status, contraceptive use, socio-economic status/employment) were significantly linked with the receipt of a greater number of ANC components, highlighting the importance of women's empowerment in accessing quality maternity care.[66] |
| Nisingizwe et al 2020 [67] | Rwanda | Quantitative | To explain perceived barriers to accessing quality ANC | perceived barriers | 64% of women who perceived to have barriers to health care had inadequate ANC visits. |
| Tato et al [68] | Zambia | Quantitative | To assess abuse and disrespectful care on women during access to antenatal care services and its implications. | Poor recommended visits | One third (33%) of the participants attended less than half of the recommended ANC visits. significant association between; physical abuse; not being allowed to assume position of choice during examination; not having privacy during examination and antenatal care service utilization. The difference in the logs of expected count on the number of ANC visits is lower for women who experienced lack of privacy during examinations, who discriminated based on specific attributes and who were left unattended. |

| Study | Country | Study | Aim | Quality of ANC | Key findings |
|---|---|---|---|---|---|
| Wynne et al 2020 [69] | Peru | Quantitative | To identify areas for improvement through comparison with antenatal guidelines | Poor adherence to guideline | Hand-held antenatal cards revealed that 52.9% of participants began their ANC the first trimester. 42.1% attended appointments at recommended gestational ages and no women received all recommended ANC services. Most women received information about identifying complications in pregnancy and health and lifestyle topics. |
| Young et al 2020.[70] | Tanzania | Quantitative | To determine quality of ANC and attendance and services | Factors of ANC quality | The most variance in the QoC index included: gave urine; gave blood; and blood pressure measured. factors associated with higher QoC included: ANC at a hospital; older age; higher level of education; working outside the home; higher socioeconomic status; and having lower parity. |
| Lire et al 2020 [71] | Southern Ethiopia | Quantitative | To assess ANC service satisfaction and associated factors among pregnant women | Satisfaction of quality ANC services | 79.2% were satisfied with the ANC service. Respondents who had received iron tablets were 3.2 times more likely to be satisfied than their counterparts. Pregnant women who were counselled on HIV infection and its testing were 4.3 times more likely to be satisfied, women who waited ⩽30 minutes and who received information on foetal movement were satisfied. |
| Ahinkorah et al 2021 [72] | Cameroon | Quantitative | To examines the factors associated with the number and timing of ANC visits | Low recommended visits and timely visits | The proportions of women who had ≥8 ANC visits and first ANC visit at ≤3 months gestation was 6.3% and 35.6% respectively. Women aged 35–39 at childbirth, middle wealth quintile women, women whose husbands had higher education were more likely to have ≥8 ANC visits. Early timing of first ANC visit was low among women with birth order 3–4. The likelihood of having early ANC visits was high among women whose pregnancies were intended, the richest women and women whose husbands had higher education. |
| Anik et al [73] | South Asian (SA) region | Quantitative | To explain the measurement of adequacy of ANC | Inadequate ANC visits. Empowerments improve quality | Only 30% women received adequate ANC in SA, ranging from 8.4% in Afghanistan to 39.8% in Nepal. The poor utilisation of adequate ANC services was most prevalent among the women residing in rural areas and that of poor families as well as low empowerment status in SA countries. Conversely, highly empowered but poor was positive association with adequate ANC services. |
| Anindya et al 2021.[74] | 39 LMICs | Quantitative | To assess socioeconomic inequalities in effective coverage ANC services | Low quality adjusted coverage | The quality-adjusted coverage of RMNCH services in 39 countries was substantially lower than service contact, and ANC outcomes. UMICs had higher effective coverage levels compared with LMICs. Socioeconomic inequalities tend to be wider when using effective coverage measurement compared with crude and service contact measurements. UMICs had a lower magnitude of inequality compared with LMICs. |

| Study | Country | Study | Aim | Quality of ANC | Key findings |
|---|---|---|---|---|---|
| Arroyave et al 2021 [75] | (LMICs) | Quanti tative | To create and validate a new indicator for ANC. | ANCq | The overall mean of ANCq was 6.7, ranging from 3.5 in Afghanistan to 9.3 in Cuba and the Dominican Republic. the ANCq was inversely associated with neonatal mortality. |
| Ayalew et al 2021 [76] | Ethiopia | Quanti tative | To assess women's satisfaction and its associated factors with ANC services. | Factors associate d satisfaction of care | 53.8% of women were satisfied with the ANC services. Age of mothers, advice on danger signs in pregnancy, previous ANC visits, respectful maternity care, and planned pregnancy were significantly associated with women's satisfaction with ANC services. |
| Bobo et al [77]. | East Africa | Quanti tative | To assesses inequalities in the use of quality ANC in nine East African countries using | Low 4ANCvisits and interventions | About 54.4% had 4+ ANC contacts, but only 21% reported receiving all six services. The coverage of 4+ ANC and receipt of all six services was pro-rich within and across all countries. The highest inequality in 4+ANC contacts was in Ethiopia, while women in Burundi had the highest inequality in coverage of all six services. Higher education levels and media exposure were predictors of service uptake, while women who had unintended pregnancies were less likely to make four or more ANC contacts and receive six services. Most women received low-quality ANC across all countries and socioeconomic levels, even when they managed to have adequate contact with providers. Those women who received quality care had primary or higher education, reside in urban areas or had plans to have a child. |
| Kedir et al [78] | Ethiopia | Quanti tative | To assess the iron-folic acid supplements and associated factors | Compliance of iron uptake | Pill count compliance rate was found to be (154) 38.3%. In addition, pregnant mothers who had anaemia in their previous pregnancy, counselling on iron-folate supplements, awareness of the benefit of the iron-folate supplements and being a member of the Health Development Army were significantly associated with compliance with iron-folate supplement. |
| Khatri et al [79] | Nepal | Quanti tative | To investigate the determinants of utilisation of technical quality MNH services | Nurse midwifes improved quality | Women utilised quality ANC services if they attended facilities with better HF capacity. Women utilised better technical quality ANC provided by nursing staff, and from staff supervised by a higher authority. |
| Kumbeni et al 2021[80] | Ghana | Quanti tative | To investigate the relationship between time spent during the first ANC | Poor compliance of recommended visits | The proportion of mothers who attained eight or more ANC contacts during pregnancy was 31.2%. Spending 20 minutes or more during the first ANC contact was associated with 2.07 times of having eight or more ANC contacts. Mothers who received at least a home visit from skilled providers during pregnancy had higher eight or more ANC contacts. |

| Study | Country | Study | Aim | Quality of ANC | Key findings |
|---|---|---|---|---|---|
| Motlagh.[81] | Iran | Quantitative | to determine the factors affecting ANC quality in the first level of network system | High contact but inadequate visit | Almost universal received prenatal care but half of them received inadequate care. associated factors were women's education level, marriage age, husband's education, husband's employment status, age in the first pregnancy, the time intervals among pregnancies, place of receiving care and health care provider. |
| Nisha et al 2021 [82] | Bangladesh | Qualitative | to explore the socio-cultural factors that influence women's early and adequate utilisation of ANC | Barriers of early initiation of ANC | Early ANC visit was not a priority, women’s lack of awareness about the appropriate timing of the first ANC contact, lack of decision-making autonomy and fear of caesarean section were the major barriers to early and adequate ANC utilisation, superstitions around pregnancy in rural settings which prevented women seeking early and adequate ANC and led them to seek care from traditional care. |
| Olorunsaiye et al 2021 [83] | West and Central Africa | Quantitative | To examine associated factors with the quality of ANC. | Inadequate ANC visits | Less than one-fourth of women received high-quality ANC including poor uptake of counselling on malaria prophylaxis and testing for HIV, living in a poor community was associated with reduced odds of receiving high-quality ANC. |
| Tadele et al 2021 [84] | Ethiopia | Quantitative | To describe the status of the adequacy of prenatal care | Inadequate services | Of the total pregnant women 44.21 % attended enough visits, 84.10 % had early visits, and 42.03 % received sufficient services. Adequate care among women residing in urban areas, attending primary and secondary education, and participating in one to five networks. |
| Feijen-de Jong et al 2012 [85] | High income countries | Review | To review the evidence for the determinants of prenatal healthcare utilization in high-income countries | Determinants of inadequate care | Low maternal age, low educational level, non-marital status, ethnic minority, planned pattern of prenatal care, hospital type, unplanned place of delivery, uninsured status, high parity, no previous premature birth, and late recognition of pregnancy were identified as individual determinants of inadequate use. Contextual determinants - living in neighbourhoods with higher rates of unemployment, single parent families, medium–average family incomes, low-educated residents, and indigenous were associated with inadequate use or late visit, and inadequate use was more likely among women who smoked during pregnancy. |
| Lassi et al [86] | Many countries | Review | To review the effectiveness of care delivered through community level inputs for improving MNH outcomes. | Task shifting reduce ANC hospitalisation. home visitation improved ANC | Task shifting to midwives and CHWs, home visitation significantly improved ANC, tetanus immunization coverage, referral and early initiation of breast feeding with reductions in antenatal hospital admission. Training of TBAs as a part of community-based intervention package significantly impacts referrals. Home visitation, community mobilization and training of CHWs and TBA have the maximum potential to improve a range of maternal and newborn health outcomes. |

| Study | Country | Study | Aim | Quality of ANC | Key findings |
|---|---|---|---|---|---|
| Moron et al [87] | Worldwide | Review | To describe indicators used for the assessment of ANC quality worldwide under the WHO framework | Less focused on promotion in ANC studies | 8.7% of the articles reported healthy eating counselling and 52.2% iron and folic acid supplementation. The evaluation indicators on maternal and fetal interventions were syphilis testing (55.1%), HIV testing (47.8%), gestational diabetes mellitus screening (40.6%) and ultrasound (27.5%). Essential ANC activities assessment ranged from 26.1% report of fetal heart sound, 50.7% of maternal weight and 63.8% of blood pressure. Tetanus vaccine was reported in 60.9% of the articles. Interventions performed by health services to improve use and quality of ANC care, maternal and fetal health promotion, and the number of visits to the ANC. |
| Rowe et al [88] | Many countries | Review | To review the evidence on measurement properties of prenatal care utilization indices | Adequacy Perinatal Care Utilization Index and the Kessner Index | Most of the indices of adequacy of prenatal care currently used in research and clinical practice have been evaluated. Evidence about the responsiveness to change of these indices is absent from these evaluations. The Adequacy Perinatal Care Utilization Index (APNCUI) and the Kessner Index are supported by moderate evidence regarding. |
| Ansu et al [89] | SSA | Scoping review | To review mapped evidence on managers' and providers' perspective. | Perception of poor maternal care | managers' and providers' perspectives provided poor quality of maternal healthcare. Factors contributing to the perception of poor maternal healthcare included: late reimbursement of funds, heavy workload of providers, lack of essential drugs and stock-out of medical supplies, lack of policy definition, OOP payment, and inequitable distribution of staff. |
| Arroyave et al [90] | Many LMICs | Systematic review | To conduct a global analysis of socioeconomic inequalities in ANC using national surveys from LMICs | Inequalities in quality care between and within countries | Higher ANCq scores were observed among women living in urban areas, with secondary or more level of education, belonging to wealthier families and with higher empowerment in nearly all countries. In addition, countries with higher ANCq mean presented lower inequalities; while countries with average ANCq scores presented wide range of inequality, with some managing to achieve very low inequality. |
| Lu et al [91]. | Many countries | Review | To examine innovative strategies, including the use of health information technology (health IT)] | health information technology can be used to reduce disparities in prenatal care quality | Strategies used to (a) increase access to timely prenatal care, (b) improve the content of prenatal care, and (c) enhance the organization and delivery of prenatal care. (a) increase consumer awareness about the importance of preconception and early prenatal care, facilitate spatial mapping of access gaps, and improve continuity of patient records; (b) support collaborative quality improvement, facilitate performance |

| Study | Country | Study | Aim | Quality of ANC | Key findings |
|---|---|---|---|---|---|
| | | | | | measurement, enhance health promotion, assist with care coordination, reduce clinical errors, improve delivery of preventive health services, provide decision support, and encourage completeness of documentation; and (c) support data integration and engineer collaborative innovation. |

## References


1. Magoma M, Requejo J, Merialdi M, Campbell OM, Cousens S, Filippi V: **How much time is available for antenatal care consultations? Assessment of the quality of care in rural Tanzania**. *BMC Pregnancy Childbirth* 2011, **11**:64.
2. Conrad P, De Allegri M, Moses A, Larsson EC, Neuhann F, Müller O, Sarker M: **Antenatal care services in rural Uganda: missed opportunities for good-quality care**. *Qual Health Res* 2012, **22**(5):619-629.
3. Kumbani LC, Chirwa E, Malata A, Odland JO, Bjune G: **Do Malawian women critically assess the quality of care? A qualitative study on women's perceptions of perinatal care at a district hospital in Malawi**. *Reproductive Health* 2012, **9**(1).
4. Kyei NNA, Chansa C, Gabrysch S: **Quality of antenatal care in Zambia: A national assessment**. *BMC Pregnancy and Childbirth* 2012, **12**.
5. Naariyong S, Poudel KC, Rahman M, Yasuoka J, Otsuka K, Jimba M: **Quality of antenatal care services in the Birim North District of Ghana: contribution of the community-based health planning and services program**. *Matern Child Health J* 2012, **16**(8):1709-1717.
6. Nyamtema AS, Jong AB, Urassa DP, Hagen JP, van Roosmalen J: **The quality of antenatal care in rural Tanzania: what is behind the number of visits?** *BMC Pregnancy and Childbirth* 2012, **12**.
7. Tetui M, Ekirapa EK, Bua J, Mutebi A, Tweheyo R, Waiswa P: **Quality of Antenatal care services in eastern Uganda: implications for interventions**. *Pan Afr Med J* 2012, **13**:27.
8. Atinga RA, Baku AA: **Determinants of antenatal care quality in Ghana**. *International Journal of Social Economics* 2013, **40**(10):852-865.
9. Ejigu T, Woldie M, Kifle Y: **Quality of antenatal care services at public health facilities of Bahir-Dar special zone, Northwest Ethiopia**. *BMC Health Serv Res* 2013, **13**:443.
10. Manithip C, Edin K, Sihavong A, Wahlström R, Wessel H: **Poor quality of antenatal care services-Is lack of competence and support the reason? An observational and interview study in rural areas of Lao PDR**. *Midwifery* 2013, **29**(3):195-202.
11. Bar-Zeev S, Barclay L, Kruske S, Kildea S: **Factors affecting the quality of antenatal care provided to remote dwelling Aboriginal women in northern Australia**. *Midwifery* 2014, **30**(3):289-296.
12. Duysburgh E, Williams A, Williams J, Loukanova S, Temmerman M: **Quality of antenatal and childbirth care in northern Ghana**. *Bjog* 2014, **121 Suppl 4**:117-126.
13. El Aty MAA, Meky FA, Morsy M, El Sayed MK: **Overall adequacy of antenatal care in Oman: secondary analysis of national reproductive health survey data, 2008**. *EASTERN MEDITERRANEAN HEALTH JOURNAL* 2014, **20**(12):781-788.
14. Hodgins S, D'Agostino A: **The quality-coverage gap in antenatal care: toward better measurement of effective coverage**. *Glob Health Sci Pract* 2014, **2**(2):173-181.

15. Joshi C, Torvaldsen S, Hodgson R, Hayen A: **Factors associated with the use and quality of antenatal care in Nepal: a population-based study using the demographic and health survey data**. *BMC Pregnancy Childbirth* 2014, **14**:94.
16. Majrooh MA, Hasnain S, Akram J, Siddiqui A, Memon ZA: **Coverage and quality of antenatal care provided at primary health care facilities in the 'Punjab' province of 'Pakistan'**. *PLoS One* 2014, **9**(11):e113390.
17. Saizonou J, Agueh DV, Aguemon B, Adé VM, Assavedo S, Makoutodé M: **[Quality assessment of refocused antenatal care services at the district hospital of Suru-Léré in Benin]**. *Sante Publique* 2014, **26**(2):249-257.
18. Achia TN, Mageto LE: **Individual and contextual determinants of adequate maternal health care services in Kenya**. *Women Health* 2015, **55**(2):203-226.
19. Fagbamigbe AF, Idemudia ES: **Assessment of quality of antenatal care services in Nigeria: evidence from a population-based survey**. *Reprod Health* 2015, **12**:88.
20. Kambala C, Lohmann J, Mazalale J, Brenner S, De Allegri M, Muula AS, Sarker M: **How do Malawian women rate the quality of maternal and newborn care? Experiences and perceptions of women in the central and southern regions**. *BMC Pregnancy Childbirth* 2015, **15**:169.
21. Villadsen SF, Negussie D, Gebremariam A, Tilahun A, Friis H, Rasch V: **Antenatal care strengthening for improved quality of care in Jimma, Ethiopia: An effectiveness study Health policies, systems and management**. *BMC Public Health* 2015, **15**(1).
22. Bayou YT, Mashalla YS, Thupayagale-Tshweneagae G: **The adequacy of antenatal care services among slum residents in Addis Ababa, Ethiopia**. *BMC Pregnancy Childbirth* 2016, **16**(1):142.
23. Cohen J, Golub G, Kruk ME, McConnell M: **Do active patients seek higher quality prenatal care?: A panel data analysis from Nairobi, Kenya**. *Prev Med* 2016, **92**:74-81.
24. Heredia-Pi I, Servan-Mori E, Darney BG, Reyes-Morales H, Lozano R: **Measuring the adequacy of antenatal health care: a national cross-sectional study in Mexico**. *Bull World Health Organ* 2016, **94**(6):452-461.
25. Servan-Mori E, Sosa-Rubí SG, Najera-Leon E, Darney BG: **Timeliness, frequency and content of antenatal care: which is most important to reducing indigenous disparities in birth weight in Mexico?** *Health Policy Plan* 2016, **31**(4):444-453.
26. Do M, Wang WJ, Hembling J, Ametepi P: **Quality of antenatal care and client satisfaction in Kenya and Namibia**. *INTERNATIONAL JOURNAL FOR QUALITY IN HEALTH CARE* 2017, **29**(2):183-193.
27. Fagbamigbe AF, Mashabe B, Lepetu L, Abel C: **Are the timings and risk factors changing? Survival analysis of timing of first antenatal care visit among pregnant women in Nigeria (2003-2013)**. *Int J Womens Health* 2017, **9**:807-819.
28. Koroma MM, Kamara SS, Bangura EA, Kamara MA, Lokossou V, Keita N: **The quality of free antenatal and delivery services in Northern Sierra Leone**. *Health Res Policy Syst* 2017, **15**(Suppl 1):49.
29. Leslie HH, Malata A, Ndiaye Y, Kruk ME: **Effective coverage of primary care services in eight high-mortality countries**. *BMJ Glob Health* 2017, **2**(3):e000424.
30. Leslie HH, Sun Z, Kruk ME: **Association between infrastructure and observed quality of care in 4 healthcare services: A cross-sectional study of 4,300 facilities in 8 countries**. *PLoS Med* 2017, **14**(12):e1002464.
31. Miltenburg AS, Van Der Eem L, Nyanza EC, Van Pelt S, Ndaki P, Basinda N, Sundby J: **Antenatal care and opportunities for quality improvement of service provision in resource limited settings: A mixed methods study**. *PLoS ONE* 2017, **12**(12).
32. Muchie KF: **Quality of antenatal care services and completion of four or more antenatal care visits in Ethiopia: a finding based on a demographic and health survey**. *BMC Pregnancy Childbirth* 2017, **17**(1):300.
33. Serván-Mori E, Contreras-Loya D, Gomez-Dantés O, Nigenda G, Sosa-Rubí SG, Lozano R: **Use of performance metrics for the measurement of universal coverage for maternal care in Mexico**. *Health Policy Plan* 2017, **32**(5):625-633.

34. Sharma J, Leslie HH, Kundu F, Kruk ME: **Poor Quality for Poor Women? Inequities in the Quality of Antenatal and Delivery Care in Kenya**. *PLoS One* 2017, **12**(1):e0171236.
35. Solnes Miltenburg A, van der Eem L, Nyanza EC, van Pelt S, Ndaki P, Basinda N, Sundby J: **Antenatal care and opportunities for quality improvement of service provision in resource limited settings: A mixed methods study**. *PLoS One* 2017, **12**(12):e0188279.
36. Ataguba JEO: **A reassessment of global antenatal care coverage for improving maternal health using sub-Saharan Africa as a case study**. *PLoS ONE* 2018, **13**(10).
37. Benova L, Tunçalp Ö, Moran AC, Campbell OMR: **Not just a number: examining coverage and content of antenatal care in low-income and middle-income countries**. *BMJ Glob Health* 2018, **3**(2):e000779.
38. Coley SL, Zapata JY, Schwei RJ, Mihalovic GE, Matabele MN, Jacobs EA, Anderson CK: **More Than a "Number": Perspectives of Prenatal Care Quality from Mothers of Color and Providers**. *Womens Health Issues* 2018, **28**(2):158-164.
39. Ejigu Tafere T, Afework MF, Yalew AW: **Antenatal care service quality increases the odds of utilizing institutional delivery in Bahir Dar city administration, North Western Ethiopia: A prospective follow up study**. *PLoS One* 2018, **13**(2):e0192428.
40. Katemba BM, Bwembya P, Hamoonga TE, Chola M, Jacobs C: **Demand Side Factors Associated With Quality Antenatal Care Services: A Case Study of Lusaka District, Zambia**. *Front Public Health* 2018, **6**:285.
41. Rurangirwa AA, Mogren I, Ntaganira J, Govender K, Krantz G: **Quality of antenatal care services in Rwanda: assessing practices of health care providers**. *BMC Health Serv Res* 2018, **18**(1):865.
42. Yeoh PL, Hornetz K, Shauki NIA, Dahlui M: **Evaluating the quality of antenatal care and pregnancy outcomes using content and utilization assessment**. *Int J Qual Health Care* 2018, **30**(6):466-471.
43. Afulani PA, Buback L, Essandoh F, Kinyua J, Kirumbi L, Cohen CR: **Quality of antenatal care and associated factors in a rural county in Kenya: an assessment of service provision and experience dimensions**. *BMC Health Serv Res* 2019, **19**(1):684.
44. Alyahya MS, Khader YS, Batieha A, Asad M: **The quality of maternal-fetal and newborn care services in Jordan: a qualitative focus group study**. *BMC Health Serv Res* 2019, **19**(1):425.
45. Jo Y, Alland K, Ali H, Mehra S, LeFevre AE, Pak SE, Shaikh S, Christian P, Labrique AB: **Antenatal care in rural Bangladesh: current state of costs, content and recommendations for effective service delivery**. *BMC Health Serv Res* 2019, **19**(1):861.
46. Mario DN, Rigo L, Boclin KLS, Malvestio LMM, Anziliero D, Horta BL, Wehrmeister FC, Martínez-Mesa J: **Quality of Prenatal Care in Brazil: National Health Research 2013**. *Cien Saude Colet* 2019, **24**(3):1223-1232.
47. Naz L: **The utilization of quality antenatal care in Pakistan: Analyzing health inequity in various periods**. *Journal of Population and Social Studies* 2019, **28**(1):72-88.
48. Okawa S, Win HH, Leslie HH, Nanishi K, Shibanuma A, Aye PP, Jimba M: **Quality gap in maternal and newborn healthcare: a cross-sectional study in Myanmar**. *BMJ Glob Health* 2019, **4**(2):e001078.
49. Owili PO, Muga MA, Mendez BR, Chen B: **Quality of care in six sub-Saharan Africa countries: a provider-based study on adherence to WHO's antenatal care guideline**. *Int J Qual Health Care* 2019, **31**(1):43-48.
50. Phommachanh S, Essink DR, Jansen M, Broerse JEW, Wright P, Mayxay M: **Improvement of Quality of Antenatal Care (ANC) Service Provision at the Public Health Facilities in Lao PDR: Perspective and Experiences of Supply and Demand Sides**. *BMC Pregnancy Childbirth* 2019, **19**(1):255.
51. Pugliese-Garcia M, Radovich E, Hassanein N, Campbell OMR, Khalil K, Benova L: **Temporal and regional variations in use, equity and quality of antenatal care in Egypt: a repeat cross-sectional analysis using Demographic and Health Surveys**. *BMC Pregnancy Childbirth* 2019, **19**(1):268.

52. Rios-Zertuche D, Zúñiga-Brenes P, Palmisano E, Hernández B, Schaefer A, Johanns CK, Gonzalez-Marmol A, Mokdad AH, Iriarte E: **Methods to measure quality of care and quality indicators through health facility surveys in low- and middle-income countries**. *Int J Qual Health Care* 2019, **31**(3):183-190.
53. Singh L, Dubey R, Singh S, Goel R, Nair S, Singh PK: **Measuring quality of antenatal care: a secondary analysis of national survey data from India**. *Bjog* 2019, **126 Suppl 4**:7-13.
54. Thida T, Liabsuetrakul T: **Type of health care workers and quality of antenatal and delivery care in communities of Myanmar: An experience- and vignette-based study**. *Int J Health Plann Manage* 2019, **34**(4):e1597-e1608.
55. Yakob B, Gage A, Nigatu TG, Hurlburt S, Hagos S, Dinsa G, Bowser D, Berman P, Kruk ME, Tekle E: **Low effective coverage of family planning and antenatal care services in Ethiopia**. *Int J Qual Health Care* 2019, **31**(10):725-732.
56. Adhikari M, Chalise B, Bista B, Pandey AR, Upadhyaya DP: **Sociodemographic correlates of antenatal care visits in Nepal: results from Nepal Demographic and Health Survey 2016**. *BMC Pregnancy Childbirth* 2020, **20**(1):513.
57. Albert JS, Younas A, Victor G: **Quality of Antenatal Care Services in a Developing Country: A Cross-Sectional Survey**. *CREATIVE NURSING* 2020, **26**(1):E25-E34.
58. de Andrade RB, Pirkle CM, Sentell T, Bassani D, Domingues MR, Câmara SMA: **Adequacy of prenatal care in northeast Brazil: Pilot data comparing attainment of standard care criteria for first-time adolescent and adult pregnant women**. *International Journal of Women's Health* 2020, **12**:1023-1031.
59. Benski AC, Schmidt NC, Viviano M, Stancanelli G, Soaroby A, Reich MR: **Improving the Quality of Antenatal Care Using Mobile Health in Madagascar: Five-Year Cross-Sectional Study**. *JMIR Mhealth Uhealth* 2020, **8**(7):e18543.
60. Berehe TT, Modibia LM: **Assessment of Quality of Antenatal Care Services and Its Determinant Factors in Public Health Facilities of Hossana Town, Hadiya Zone, Southern Ethiopia: A Longitudinal Study**. *Advances in Public Health* 2020, **2020**.
61. Dadras O, Dadras F, Taghizade Z, Seyedalinaghi S, Ono-Kihara M, Kihara M, Nakayama T: **Barriers and associated factors for adequate antenatal care among Afghan women in Iran; findings from a community-based survey**. *BMC Pregnancy Childbirth* 2020, **20**(1):427.
62. Defar A, Getachew T, Taye G, Tadele T, Getnet M, Shumet T, Molla G, Gonfa G, Teklie H, Tadesse A *et al*: **Quality antenatal care services delivery at health facilities of Ethiopia, assessment of the structure/input of care setting**. *BMC Health Serv Res* 2020, **20**(1):485.
63. Fauziah N, Ansariadi A, Darmawansyah D, Wahidin M, Amaliah R, Tasya Z, Annah I, Yanti IH: **Quality of antenatal care at urban and rural puskesmas (Public health center) in jeneponto regency**. *Open Access Macedonian Journal of Medical Sciences* 2020, **8**(T2):177-182.
64. Harsha Bangura A, Nirola I, Thapa P, Citrin D, Belbase B, Bogati B, B.k N, Khadka S, Kunwar L, Halliday S *et al*: **Measuring fidelity, feasibility, costs: An implementation evaluation of a cluster-controlled trial of group antenatal care in rural Nepal**. *Reproductive Health* 2020, **17**(1).
65. Hategeka C, Arsenault C, Kruk ME: **Temporal trends in coverage, quality and equity of maternal and child health services in Rwanda, 2000-2015**. *BMJ Glob Health* 2020, **5**(11).
66. Merrell LK, Blackstone SR: **Women's Empowerment as a Mitigating Factor for Improved Antenatal Care Quality despite Impact of 2014 Ebola Outbreak in Guinea**. *Int J Environ Res Public Health* 2020, **17**(21).
67. Nisingizwe MP, Tuyisenge G, Hategeka C, Karim ME: **Are perceived barriers to accessing health care associated with inadequate antenatal care visits among women of reproductive age in Rwanda?** *BMC Pregnancy Childbirth* 2020, **20**(1):88.
68. Tato Nyirenda H, Nyirenda T, Choka N, Agina P, Kuria S, Chengo R, H BCN, Mubita B: **Abuse and disrespectful care on women during access to antenatal care services and its implications in Ndola and Kitwe health facilities**. *Sex Reprod Healthc* 2020, **26**:100554.
69. Wynne SJ, Duarte R, de Wildt G, Meza G, Merriel A: **The timing and quality of antenatal care received by women attending a primary care centre in Iquitos, Peru: A facility exit survey**. *PLoS One* 2020, **15**(3):e0229852.

70. Young MR, Morof D, Lathrop E, Haddad L, Blanton C, Maro G, Serbanescu F: **Beyond adequate: Factors associated with quality of antenatal care in western Tanzania**. *Int J Gynaecol Obstet* 2020, **151**(3):431-437.
71. Lire T, Megerssa B, Asefa Y, Hirigo AT: **Antenatal care service satisfaction and its associated factors among pregnant women in public health centres in Hawassa city, Southern Ethiopia**. *PROCEEDINGS OF SINGAPORE HEALTHCARE* 2021.
72. Ahinkorah BO, Seidu AA, Budu E, Mohammed A, Adu C, Agbaglo E, Ameyaw EK, Yaya S: **Factors associated with the number and timing of antenatal care visits among married women in Cameroon: evidence from the 2018 Cameroon Demographic and Health Survey**. *J Biosoc Sci* 2021:1-11.
73. Anik AI, Islam MR, Rahman MS: **Do women's empowerment and socioeconomic status predict the adequacy of antenatal care? A cross-sectional study in five South Asian countries**. *BMJ Open* 2021, **11**(6):e043940.
74. Anindya K, Marthias T, Vellakkal S, Carvalho N, Atun R, Morgan A, Zhao Y, Hulse ES, McPake B, Lee JT: **Socioeconomic inequalities in effective service coverage for reproductive, maternal, newborn, and child health: a comparative analysis of 39 low-income and middle-income countries**. *EClinicalMedicine* 2021, **40**:101103.
75. Arroyave L, Saad GE, Victora CG, Barros AJD: **A new content-qualified antenatal care coverage indicator: Development and validation of a score using national health surveys in low- and middle-income countries**. *J Glob Health* 2021, **11**:04008.
76. Ayalew MM, Nebeb GT, Bizuneh MM, Dagne AH: **Women's Satisfaction and Its Associated Factors with Antenatal Care Services at Public Health Facilities: A Cross-Sectional Study**. *Int J Womens Health* 2021, **13**:279-286.
77. Bobo FT, Asante A, Woldie M, Hayen A: **Poor coverage and quality for poor women: Inequalities in quality antenatal care in nine East African countries**. *Health Policy Plan* 2021, **36**(5):662-672.
78. Kedir Obsa A, Tegene Y, Gebretsadik A: **Iron and Folic Acid Supplementation Compliance and Associated Factors among Pregnant Women Attending Antenatal Clinic in Shalla District, Southwest Ethiopia: A Cross-Sectional Study**. *J Nutr Metab* 2021, **2021**:6655027.
79. Khatri RB, Durham J, Assefa Y: **Utilisation of quality antenatal, delivery and postnatal care services in Nepal: An analysis of Service Provision Assessment**. *Global Health* 2021, **17**(1):102.
80. Kumbeni MT, Apanga PA, Yeboah EO, Kolog JT, Awuni B: **The relationship between time spent during the first ANC contact, home visits and adherence to ANC contacts in Ghana**. *Glob Health Action* 2021, **14**(1):1956754.
81. Motlagh ME, Torkestani F, Amiri HA, Delavar MA, Radpooyan L, Shirvani SDN: **Factors affecting the adequacy of prenatal care utilization index in the first level of network system in Iran**. *Journal of Babol University of Medical Sciences* 2021, **23**(1):76-83.
82. Nisha MK, Alam A, Rahman A, Raynes-Greenow C: **Modifiable socio-cultural beliefs and practices influencing early and adequate utilisation of antenatal care in rural Bangladesh: A qualitative study**. *Midwifery* 2021, **93**:102881.
83. Olorunsaiye CZ, Brunner Huber LR, Laditka SB, Kulkarni SJ, Boyd S: **Individual and community socioeconomic factors related to the quality of antenatal care: a multilevel analysis of West and Central Africa**. *Women Health* 2021, **61**(1):15-26.
84. Tadele A, Teka B: **Adequacy of prenatal care services and associated factors in Southern Ethiopia**. *Arch Public Health* 2021, **79**(1):94.
85. Feijen-de Jong EI, Jansen DE, Baarveld F, van der Schans CP, Schellevis FG, Reijneveld SA: **Determinants of late and/or inadequate use of prenatal healthcare in high-income countries: a systematic review**. *Eur J Public Health* 2012, **22**(6):904-913.
86. Lassi ZS, Das JK, Salam RA, Bhutta ZA: **Evidence from community level inputs to improve quality of care for maternal and newborn health: interventions and findings**. *Reprod Health* 2014, **11 Suppl 2**(Suppl 2):S2.
87. Moron-Duarte LS, Varela AR, Segura O, da Silveira MF: **Quality assessment indicators in antenatal care worldwide: a systematic review**. *INTERNATIONAL JOURNAL FOR QUALITY IN HEALTH CARE* 2019, **31**(7):497-505.
88. Rowe S, Karkhaneh Z, MacDonald I, Chambers T, Amjad S, Osornio-Vargas A, Chari R, Kumar M, Ospina MB: **Systematic review of the measurement properties of indices of prenatal care utilization**. *BMC Pregnancy Childbirth* 2020, **20**(1):171.

89. Ansu-Mensah M, Danquah FI, Bawontuo V, Ansu-Mensah P, Mohammed T, Udoh RH, Kuupiel D: **Quality of care in the free maternal healthcare era in sub-Saharan Africa: a scoping review of providers' and managers' perceptions**. *BMC Pregnancy Childbirth* 2021, **21**(1):220.
90. Arroyave L, Saad GE, Victora CG, Barros AJD: **Inequalities in antenatal care coverage and quality: an analysis from 63 low and middle-income countries using the ANCq content-qualified coverage indicator**. *Int J Equity Health* 2021, **20**(1):102.
91. Lu MC, Kotelchuck M, Hogan VK, Johnson K, Reyes C: **Innovative strategies to reduce disparities in the quality of prenatal care in underresourced settings**. *Med Care Res Rev* 2010, **67**(5 Suppl):198s-230s.
